# Clinical immunology in chromatinopathies: a scoping review

**DOI:** 10.3389/fimmu.2026.1773284

**Published:** 2026-03-26

**Authors:** Dieke Jans, Burcu Al, Arianne Bouman, Joyce M. Geelen, Tjitske Kleefstra, Katarzyna Placek

**Affiliations:** 1 Department of Human Genetics, Radboud University Medical Center, Nijmegen, Netherlands; 2 Department for Immunology & Metabolism, Life and Medical Sciences Institute (LIMES), University of Bonn, Bonn, Germany; 3 Donders Institute for Brain, Cognition, and Behavior, Radboud University Medical Center, Nijmegen, Netherlands; 4 Department of Pediatrics, Radboud Institute for Health Sciences, Radboud University Medical Center, Amalia Children’s Hospital, Nijmegen, Netherlands; 5 Center of Excellence for Neuropsychiatry, Vincent van Gogh Institute for Psychiatry, Venray, Netherlands; 6 Department of Clinical Genetics, Erasmus Medical Center (MC), Rotterdam, Netherlands

**Keywords:** autoimmunity, chromatin, epigenetics, histone modifications, hypogammaglobulinemia, immunology, infection, neurodevelopmental disorders

## Abstract

**Background:**

Chromatinopathies (CP) are a subclass of monogenic neurodevelopmental disorders caused by germline mutations in genes involved in epigenetic regulation. Symptoms may include neurodevelopmental delay, intellectual disability, autism spectrum disorder, facial dysmorphisms, growth abnormalities, and various congenital anomalies. These disorders often also present with immunopathology. However, a clear insight into immune dysregulation in CPs is currently unavailable.

**Objective:**

This scoping review aims to synthesize clinical data on anomalies of the immune system, including laboratory values and medical history of individuals with CPs, to fill knowledge gaps.

**Methods:**

Articles on immunological aspects in individuals with CPs, caused by germline mutations affecting chromatin-modifying enzymes from the “writers” group, were included. Searches were conducted using PubMed, Cochrane Library, and Web of Science until March 30, 2024 and articles after that date were added as cross-referenced.

**Results:**

101 articles covering 715 individuals with 21 different syndromes were included. In all CPs, frequent infections, mainly of the respiratory tract, were reported. Moreover, common immunopathology included immunodeficiency and immune thrombocytopenic purpura. Immunologic laboratory results were available for a total of 183 individuals with ten different syndromes. Hypo/a-gammaglobulinemia was present in the majority of these individuals. The most characterized and affected syndromes were ICF syndrome type 1 and Kabuki syndrome type 1.

**Conclusion:**

Despite common immune manifestations, systematic laboratory tests on immune characteristics are not commonly performed for the majority of CP cases. To better understand the role of immune dysfunction in the pathology of CPs and offer treatment strategies, standardized guidelines for clinicians need to be established.

**Systematic Review Registration:**

https://inplasy.com/wp-content/uploads/2025/12/INPLASY-Protocol-8609.pdf, identifier INPLASY2025120066.

## 1 Introduction

Chromatinopathies. Chromatinopathies (CPs) represent a subclass of monogenic neurodevelopmental disorders (mNDD) caused by germline mutations in genes involved in epigenetic regulation (1). CPs are also referred to as Mendelian disorders of the epigenetic machinery (MDEMs). Collectively, MDEMs are responsible for a common etiology with intellectual disability and growth deviations, mostly resulting from a loss of function of affected proteins. Components of the epigenetic machinery involved in MDEM can be categorized into four groups: writers, erasers, readers, and remodelers (2). Causative mutations lead to loss of function of those specific epigenetic components, resulting in epigenetic abnormalities, which lead to phenotypic deviations. Therefore, a proper balance between writers, which add chromatin modifications, and erasers, which remove them, must be maintained, as imbalances in epigenetic mechanisms can result in disorders (2–4).

Clinic. Clinical manifestations demonstrate heterogeneity due to different underlying genetic mutations and different effects on chromatin regulation. Common clinical symptoms include neurodevelopmental delay, intellectual disability, autism spectrum disorder, and facial dysmorphism (5). In addition, immune dysregulation poses another important clinical symptom in mNDDs belonging to the subclass CP (1). Some CPs have already been classified as primary inborn errors of immunity (6). Immunopathology in CPs likely results from genes involved in CPs also being involved in immunological mechanisms (1). Chromatin accessibility, histone modifications, and DNA methylation are involved in the development and function of various immune cells, including B lymphocytes (7–9); T lymphocytes (10); and myeloid cells (11, 12). Subsequently, immunopathologies may manifest more prominently in CPs than anticipated so far.

Epigenetic regulation. The epigenome has been suggested as the key link between environmental factors and the genome. Representing heritable alterations to gene expression without modification of the DNA sequence, it is pivotal for chromatin regulation, thereby controlling DNA accessibility and gene transcription (13). Within the nucleus, DNA is organized into chromatin, comprising repeated nucleosomal units. Each unit consists of approximately 150 base pairs of DNA wrapped around a core of 8 histone (H) proteins (two copies of each H2A, H2B, H3, and H4). Chromatin fibers exhibit dynamic flexibility and looping, facilitating communication between DNA sequences and their regulatory elements (13). Epigenetic mechanisms include DNA methylation, histone modifications, chromatin accessibility, non-coding RNAs, and DNA looping, which orchestrate the dynamic organization of chromatin, forming its epigenome (13). Chromatin structure, which is modulated by these various modifications, provides a cell-specific road map for the transcriptional machinery to target genes destined to be activated or suppressed. For example, gene loci located within open chromatin, which is enriched in acetylated histones, methylated lysine (K) 4 on H3, and long-range DNA interactions, are easily accessible to transcriptional machinery and can be readily transcribed. Furthermore, while H3K4 methylation is mainly found on promoter genes and associated with gene activation, H3K36 methylation is distributed along gene bodies and is essential for transcription, recombination, and DNA damage response. Gene loci that are located in closed chromatin enriched in methylated DNA (14) or suppressive histone marks such as H3K27 trimethylation or H3K9 dimethylation and that have few interactions with other DNA regions remain suppressed. With tremendous recent developments in technologies to study and to edit the epigenome, we are gaining more comprehension of the highly complex epigenetic regulation (14).

The immune system. The immune system has evolved to protect the host from various diseases and to maintain the homeostasis in the body. Its proper functioning relies on complex interactions between numerous cell types mounting innate (early, non-specific) and adaptive (late, antigen-specific) responses to invading pathogens or stress signals. Innate immunity is represented by the phagocytic system, complement, acute-phase response, natural killer (NK) cells, and dendritic cells, providing immediate host defense. Adaptive immunity is represented by B and T lymphocytes providing antigen-specific responses (15, 16). All immune cell lineages originate from hematopoietic stem cells (HSC) (17). While B cells and myeloid cells mature in the bone marrow, T lymphocyte progenitors migrate to the thymus, where they undergo further specification and become mature T cells. Dysregulation of the immune system’s development and function has various consequences for human health.

Epigenetic regulation of the immune system.Epigenetic modifications are functionally connected to the differentiation, activation, function, and exhaustion of immune cells (13) (**Supplementary Figure 1**). For example, hematopoiesis is accompanied by gradual reorganization of the chromatin landscape, where global changes in DNA methylation, histone modifications, and chromatin accessibility result in *de novo* establishment of lineage-specific regulatory elements (18–20). Therefore, it comes as no surprise that mutations or loss of epigenetic regulators lead to abnormal hematopoiesis (19).

Later stages of immune cell development are also regulated by epigenetic machinery. The variable-diversity-joining (V-D-J) recombination and allelic exclusion, which drive selective T and B cell receptor expression, are tightly regulated by chromatin localization, DNA methylation, and activating histone marks such as acetylation and H3K4 trimethylation (21–26). Furthermore, global DNA methylation patterns are progressively lost as naive B cells transition to plasma cells (13). B cell activation and function also depend on DNA methylation and accessibility, histone modifications, and non-coding RNAs that sustain the transcriptional identity of the different B cell subsets (27). Similarly, during T cell development in the thymus and differentiation in the periphery, the epigenetic landscape remarkably changes (28–31). As the thymocytes progress through CD8^-^CD4^-^ double negative (DN) stages via CD4^+^CD8^+^ double positive (DP) to mature CD4^+^ or CD8^+^ single positive (SP) stages, the chromatin accessibility landscape undergoes consecutive remodeling with major changes marking T cell lineage commitment (32). Further, the fate decision of DP T cells to become either CD4^+^ or CD8^+^ SP T cells is, among others, driven by CD4 and CD8 regulatory elements, which are epigenetically regulated (33, 34). In the periphery, as a naïve CD4^+^ T cell undergoes differentiation to either IFN-γ-producing T helper type 1 (Th1) or IL-4-producing Th2 cells, the expression of the signature cytokines and master transcription factors is regulated by chromatin structure, where Th1-specific loci become enriched in activating histone marks while Th2-specific genes are enriched in suppressive histone marks in Th1 cells and vice versa (29, 32, 33, 35).

Regulatory T (Treg) cells are a subpopulation of CD4^+^ T cells that restrain immune response. One of the most established epigenetic mechanisms in maintaining Treg cell identity is demethylation of the *FOXP3* locus, which enables the stable expression of the FOXP3 gene, a master regulator of Treg cell function (36, 37). But the involvement of epigenetic mechanisms in Treg cell development and function is more complex than just the DNA methylation status of the *FOXP3* locus. A series of studies unraveled epigenetic events leading to Treg cell differentiation from thymic precursors and from naïve CD4^+^ T cells in the periphery as well as the tissue-specific epigenetic signatures of Treg cells (38, 39). Epigenetic mechanisms also enable a plasticity of functional responses of immune cells. Genes encoding master transcription factors regulating naïve CD4^+^ T cell commitment to either Th1, Th2, Th17, or Treg cells are found to carry bivalent chromatin marks such as H3K4me3 and H3K27me3 in the opposite lineages. This poised chromatin state allows fast activation of gene expression and therefore transition from one cell type to another under specific conditions, as it has been shown, for example, for Treg transitioning to Th1 or Th17 phenotype (35, 40). Similarly, naïve CD8^+^ T cell differentiation into effector cells is accompanied by global changes in DNA methylation, chromatin accessibility, and permissive H3K4me3 and repressive H3K27me3 distribution (41–44). For example, genes related to proliferation and differentiation are marked by both H3K4me3 and H3K27me3 in naïve CD8^+^ T cells but lose the repressive H3K27me3 mark in effector cells. Yet, genes important for effector functions lost suppressive DNA methyation concomitantly gaining permissive H3K4me3 during the transition from naïve to effector cells (43). Altogether, epigenetic modifications are essential for differentiation and activation of B and T cells.

Epigenetic regulation of innate immune cell function has also been well studied. Similar to B cells and T cells, active DNA demethylation accompanies monocyte differentiation to macrophages (45). DNA regions that become demethylated, nucleosome-free and gain activating histone marks, are enriched in genes related to macrophage function. Moreover, a seminal study mapping four histone modifications—H3K27ac, H3K4 mono-, di-, and tri-methylation, and chromatin accessibility across macrophages from seven different tissues—revealed that the tissue local environment has an impact on the chromatin landscape of macrophages, adjusting their transcriptional program to perform microenvironment-specific functions (46). Macrophage activation by pro-inflammatory or infectious agents reshapes the epigenetic landscape, with DNA regions involved in the pro-inflammatory response losing repressive marks and gaining activating marks (11, 47). Furthermore, immune memory responses of adaptive immune cells and trained immunity and tolerance induction in innate immune cells also rely on epigenetic reprogramming (48, 49). Thus, epigenetic processes are entangled in the immune system’s maturation and function, and their disruption often leads to immunopathology.

**Figure 1:**
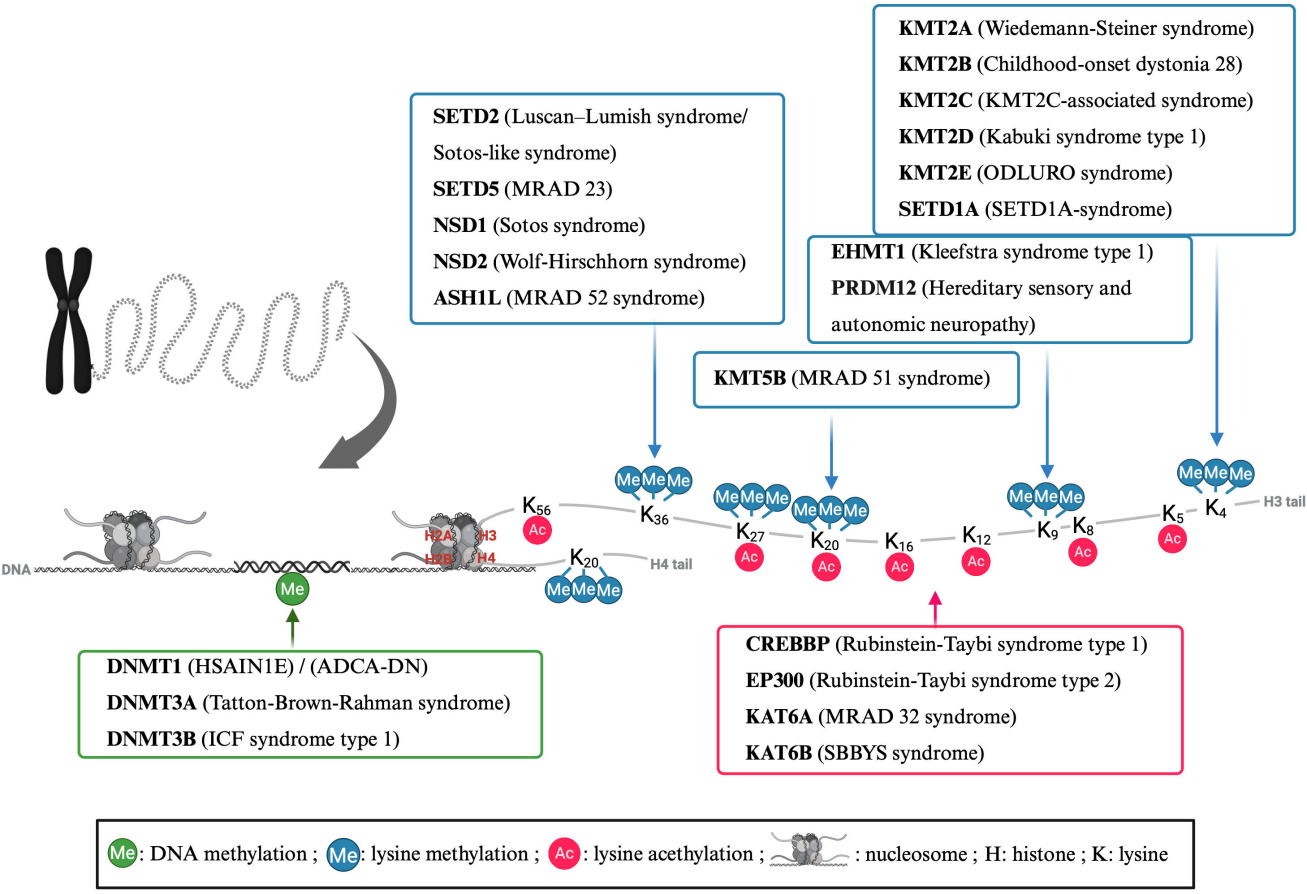
Epigenetic enzymes from the “writer” category implicated in chromatinopathies are summarized along with their target residues and associated syndromes. Key chromatin writer enzymes, including DNA methyltransferases (DNMTs), histone methyltransferases (KMTs), and histone acetyltransferases (KATs), are mapped together with their specific modification targets on DNA or histone tails. Each enzyme is linked to its corresponding epigenetic mark: DNA methylation (in green), histone lysine methylation (in blue), or histone lysine acetylation (in magenta), with arrows indicating the modified residue (e.g., H3K4, H3K27, H4K20). Distinct chromatinopathies resulting from disrupted deposition of these marks are also highlighted. ADCA-DN, autosomal dominant cerebellar ataxia, deafness, and narcolepsy; ASH1L, absent, small, or homeotic 1-like; CREBBP, CREB-binding protein; EHMT, euchromatic histone methyltransferase; EP300, E1A-associated protein p300; HSAIN1E, hereditary sensory and autonomic neuropathy 1E; ICF, immunodeficiency, centromeric region instability, facial anomalies; MRAD, intellectual disability autosomal dominant; NSD, nuclear receptor-binding SET domain protein; ODLURO, O’Donnell-Luria-Rodan; PRDI-BF1, positive regulatory domain I binding factor 1; PRDM12, PRDI-BF1 and RIZ domain-containing protein; RIZ, retinoblastoma-interacting zinc-finger; SBBYS, Say-Barber/Biesecker/Young-Simpson syndrome; SET, Su(var), Enhancer of zeste, Trithorax; SETD, SET domain-containing protein. Created with Biorender.

Aim. A comprehensive overview of immunopathology in mNDDs belonging to the subclass CP is currently unavailable. Therefore, a scoping review is conducted to systematically synthesize available clinical and immunological data about individuals with mNDDs belonging to the subclass CP. Due to the feasibility, we focused on CPs caused by mutations in epigenetic writers, which constitute the biggest group of CPs and have been most characterized in terms of immunopathology (**Figure 1**) (4). This includes laboratory data, infection history, and other manifestations of immunopathology, aiming to identify patterns and fill knowledge gaps.

## 2 Methods

### 2.1 Registration

This systematic review was registered in the INPLASY database (registration number: INPLASY2025120066) and is reported in accordance with PRISMA 2020 guidelines.

### 2.2 Research strategy

**Figure 2:**
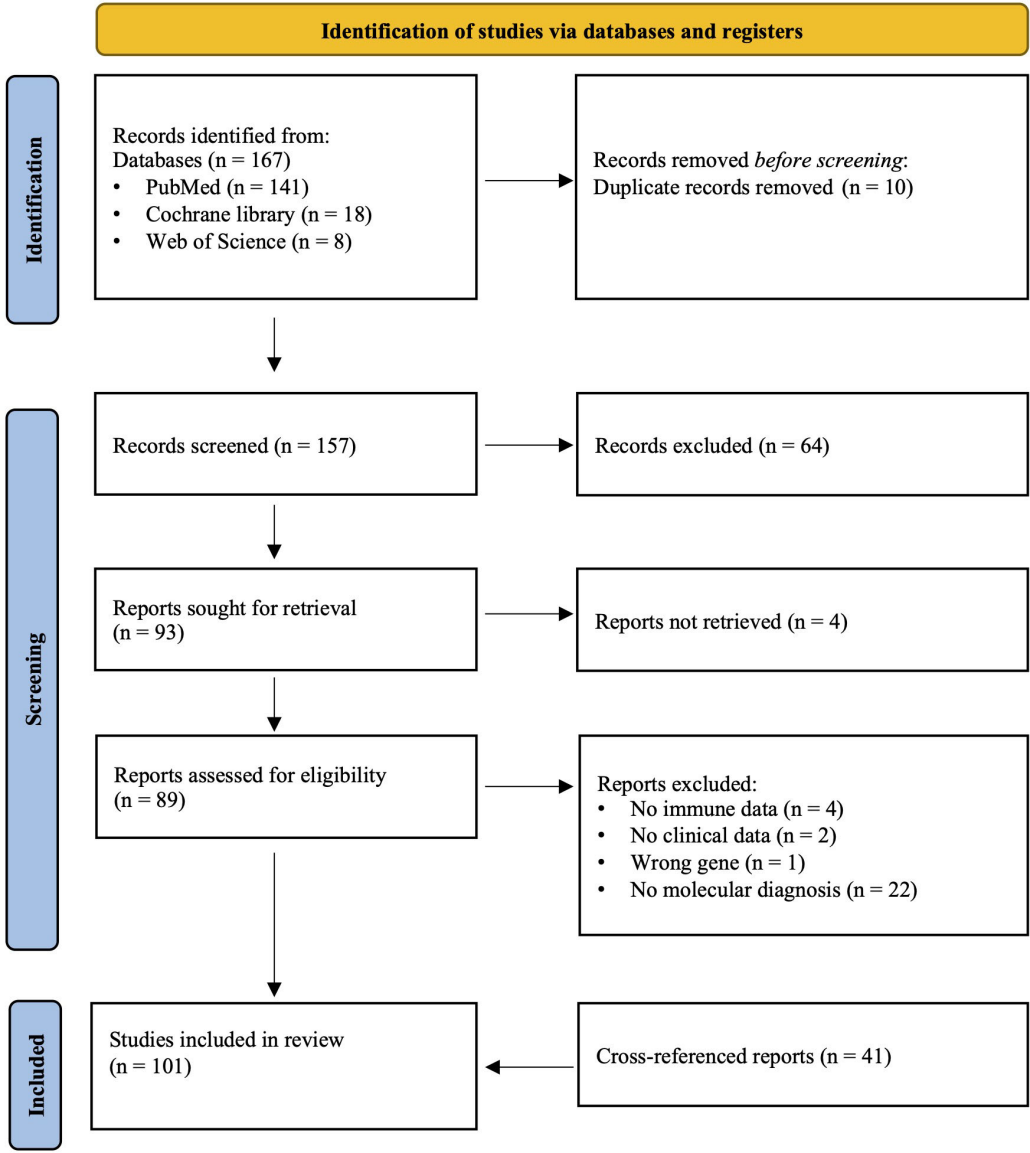
The PRISMA 2020 flow diagram outlines the study selection process for this review. Systematic identification, screening, and inclusion of studies in accordance with PRISMA 2020 guidelines are outlined. A total of 167 records were identified through database searches. After removal of duplicate records, 157 were screened for relevance. Following full-text screening, 60 studies met the inclusion criteria together with 41 cross-referenced reports, resulting in 101 articles incorporated into the final review.

To date there are 179 syndromes classified as CPs (1). In this scoping review, we focus on the “writer” category of epigenetic enzymes, the enzymes that imprint epigenetic marks on chromatin (3). The search of articles was performed using the Preferred Reporting Items for Systematic Reviews and Meta-Analysis (PRISMA) extension for scoping reviews (50). PubMed, Cochrane Registry, and Web of Science were searched to identify relevant articles. The detailed search strategy is provided in **Supplementary Table 1**. First, the comprehensive search strategy was conducted. Secondly, individual search strategies were performed for each gene that was not represented in the initial results. This step excluded the immunology-specific terms from the search strategy and included manual full-text screening of all results for immunological data. To maintain feasibility, the study exclusively included genes from the writer category of the epigenetic machinery (2–4). Forty-one additional references were added through cross-referencing (**Figure 2**).

### 2.3 Screening

After completing the search, articles were screened by evaluation of titles and abstracts first, followed by the evaluation of the full text, considering inclusion and exclusion criteria. Articles were included if they reported data on immunological aspects – immunoglobulin (Ig) levels or lymphocyte counts and/or infection history or immunopathology – in individuals with mNDDs belonging to the subclass CP caused by mutations in epigenes belonging to the “writer” category, were written in English, published in peer-reviewed journals, and involved human individuals (3). Articles were excluded if they regarded animal studies, cell studies, or cancer studies; full text was unavailable in English; or if they did not provide clinical information about the immune system in individuals with mNDDs belonging to the subclass CP and no molecular diagnosis of patients was performed.

### 2.4 Data collection

Primarily, data was collected on the number of individuals per article, age, location of medical center, gene, genetic variant, protein variant consequence, and functional consequence. Furthermore, levels of Igs (IgG, IgG1, IgG2, IgG3, IgG4, IgA, IgM) and frequencies of lymphocytes (T cells: CD3^+^, CD3^+^CD4^+^, CD3^+^CD8^+^, B cells: CD19^+^, total memory CD19^+^CD27^+^, memory IgD^+^CD27^+^, switched memory IgD^-^CD27^+^, plasma cells IgM^-^CD38^++^, naive IgM^+^/IgD^+^, CD21^low^CD38^low^, NK cells: CD16^+^/CD56^+^) were recorded. Moreover, data on infection history, autoimmunity, and other immunopathology was recorded. In cases where the consequence of genetic variants was not mentioned or unclear, this was further evaluated by use of ClinVar (www.ncbi.nlm.nih.gov/clinvar/) and Alamut (www.sophiagenetics.com/platform/alamut-visual-plus/).

### 2.5 Data analysis

Following data collection, descriptive statistics were performed to evaluate frequencies of lymphocytes and concentrations of Igs, as well as prevalence of infections and immunopathology. Absolute lymphocyte counts were noted and manually calculated if only percentages were given for subpopulations but the absolute total lymphocyte count was provided. All lymphocyte values were manually compared to the age-dependent reference values from Comans-Bitter et al. (51). However, these reference values could not be used in cases where only percentages for subpopulations were provided. In these cases, the conclusions of the article itself were used instead. Ig values were compared with Sanquin age-dependent references (www.sanquin.org) (52). The reference values are attached in **Supplementary Table 2**. IBM SPSS version 29 and GraphPad Prism version 10.1.2 were used for data analysis.

## 3 Results

### 3.1 Inclusion of articles

A total of 167 articles was identified from the search strategy of databases Pubmed, Cochrane Library, and Web of Science. After removal of duplicates, 157 articles were eligible for title and abstract screening. Based on the title and abstract, 64 articles were excluded, resulting in 93 articles eligible for full-text screening. Of these, 33 were excluded for the following reasons: inability for retrieval (n=4), no relevant immunological data (n=4), no clinical data (n=2), no molecular diagnosis (n=22), and wrong gene mutation (n=1). Consequently, the remaining 60 articles were considered eligible to be included in the scoping review. Additionally, we identified 41 eligible articles via cross-reference, making the total 101 revised articles (**Figure 2**).

### 3.2 Participants

**Figure 3:**
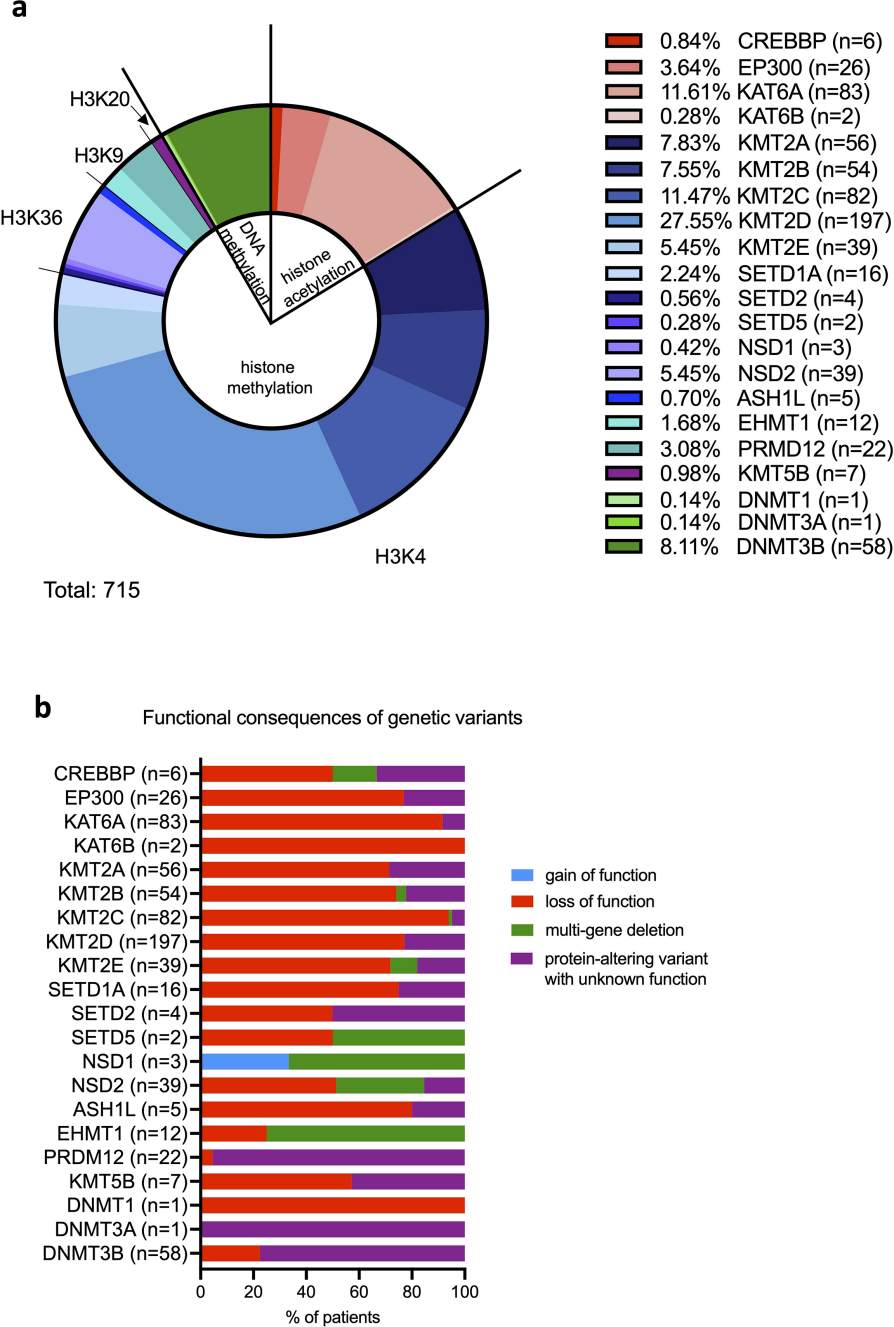
Distribution of genetic mutations and the corresponding genetic variants among the included 715 cases. **(A)** Relative frequencies of each chromatinopathy across the 715 cases. **(B)** Functional consequences of the identified genetic variants are categorized as gain of function, loss of function, multi-gene deletion, or protein-altering variant with unknown function among 715 cases. ASH1L, absent, small, or homeotic 1-like; CREBBP, CREB-binding protein; DNMT, DNA methyltransferase; EHMT, euchromatic histone methyltransferase; EP300, E1A-associated protein p300; KAT, lysine acetyltransferase; KMT, lysine methyltransferase; NSD, nuclear receptor-binding SET domain protein; PRDI-BF1, positive regulatory domain I binding factor 1; PRDM12, PRDI-BF1 and RIZ domain-containing protein; RIZ, retinoblastoma-interacting zinc-finger; SET, Su(var), Enhancer of zeste, Trithorax; SETD, SET domain-containing protein.

In total, 715 individuals with a molecularly confirmed genetic diagnosis were included in this study, comprising individuals with a germline mutation in epigenetic enzymes from the “writers” category according to Fahrner and Bjornsson 2019 (3): 1) histone methyltransferases (KMT): euchromatic histone methyltransferase (EHMT) EHMT1 (Kleefstra syndrome type 1, n=12) (53–59), KMT2A (Wiedemann-Steiner syndrome, n=56) (60–69), KMT2B (Childhood-onset dystonia 28 (DYT28), n=54) (70, 71), KMT2C (KMT2C-associated syndrome, n=82) (72–74), KMT2D (Kabuki syndrome type 1, n=197) (75–86), KMT2E (O’Donnell-Luria-Rodan (ODLURO) syndrome, n=39) (87, 88), ASH1-like (ASH1L) histone methyltransferase mutations (intellectual disability autosomal dominant (MRAD) 52 syndrome, n=5) (89), Nuclear receptor-binding SET domain protein (NSD) NSD1 (Sotos syndrome, n=3) (90, 91), NSD2 (Wolf-Hirschhorn syndrome, n=39) (92–99), PRDI-BF1 and RIZ domain-containing protein (PRDM) PRDM12 (Hereditary sensory and autonomic neuropathy, n=22) (100, 101), SET domain-containing protein (SETD) SETD1A (SETD1A-syndrome, n=16) (102, 103), SETD2 (Luscan–Lumish syndrome/Sotos-like syndrome, n=4) (104, 105), SETD5 (MRAD 23 syndrome, n=2) (106, 107), KMT5B mutations (MRAD 51 syndrome, n=7) (89); 2) histone acetyltransferases (KAT): CREB-binding protein (CREBBP) (Rubinstein-Taybi syndrome type 1, n=6) (108–119), E1A-associated protein p300 (EP300) (Rubinstein-Taybi syndrome type 2, n= 26) (120–130), KAT6A (MRAD 32 syndrome, n=83) (131–135), KAT6B (Say-Barber/Biesecker/Young-Simpson syndrome (SBBYS), n=2) (136); and 3) DNA methyltransferases (DNMT): DNMT1 (Hereditary sensory and autonomic neuropathy 1E (HSAIN1E)/Autosomal dominant cerebellar ataxia, deafness, and narcolepsy (ADCA-DN), n=1) (137), DNMT3A (Tatton-Brown-Rahman syndrome, n=1) (138), and DNMT3B (Immunodeficiency, centromeric region instability, facial anomalies (ICF) syndrome type 1, n=58) (139–154) (**Figure 3A**; **Supplementary Table 3**). Our search strategy has not identified suitable articles describing patients with EZH2 mutations (Weaver syndrome), PRMD16 mutations (dilated cardiomyopathy), and PRMD5 mutations (Brittle cornea syndrome), but we included patients with mutations in the *SETD1A* gene coding for histone (H) 3 lysine (K) 4 methyltransferase, which was not mentioned by Fahrner and Bjornsson 2019 (3, 155). Considering genetic variants, most individuals presented with a variant resulting in loss-of-function (n=499), followed by a protein-altering variant with unknown function (n=182), multi-gene deletion (n=33), and gain-of-function (n=1) (**Figure 3B**).

#### 3.2.1 Recurrent infections

**Figure 4:**
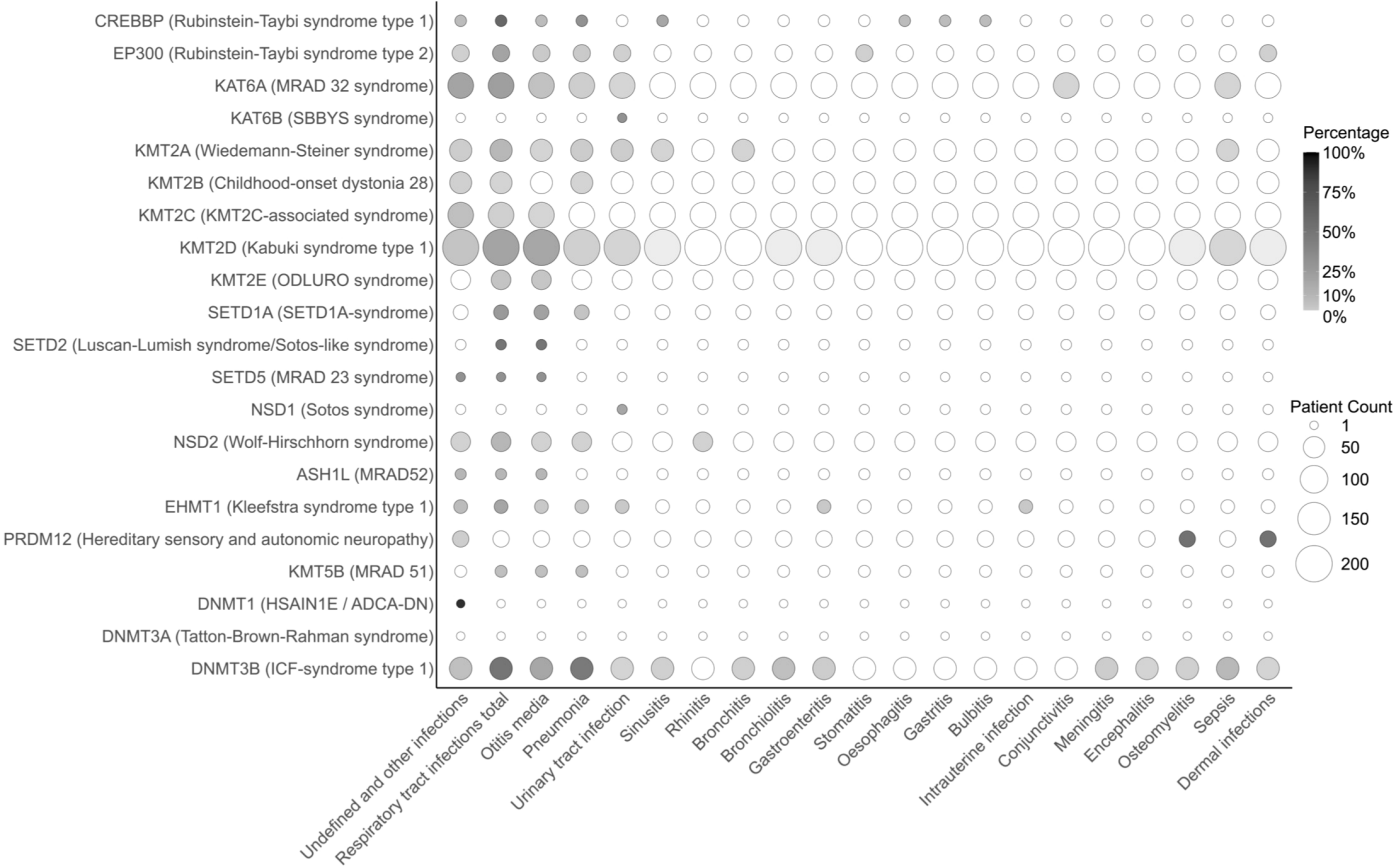
Prevalence of infections across chromatinopathies resulting from mutation in epigenetic writers. The bubble plot depicts specific types of infections on the x-axis in different chromatinopathies listed on the y-axis. The number of reported patients is illustrated as bubble sizes, whereas the percentage of incidences within each syndrome is displayed as bubble shading. ADCA-DN, autosomal dominant cerebellar ataxia, deafness, and narcolepsy; ASH1L, absent, small, or homeotic 1-like; CREBBP, CREB-binding protein; DNMT, DNA methyltransferase; EHMT, euchromatic histone methyltransferase; EP300, E1A-associated protein p300; HSAIN1E, hereditary sensory and autonomic neuropathy 1E; ICF, immunodeficiency, centromeric region instability, facial anomalies; KAT, lysine acetyltransferase; KMT, lysine methyltransferase; MRAD, intellectual disability autosomal dominant; NSD, nuclear receptor-binding SET domain protein; PRDI-BF1, positive regulatory domain I binding factor 1; PRDM12, PRDI-BF1 and RIZ domain-containing protein; RIZ, retinoblastoma-interacting zinc-finger; SBBYS, Say-Barber/Biesecker/Young-Simpson syndrome; SET, Su(var), Enhancer of zeste, Trithorax; SETD, SET domain-containing protein.

Recurrent infections are common in children, often being a part of normal childhood development. However, they can also be an indication of an immunodeficiency or chronic condition or could be due to the structural abnormality. Infections were reported in 37% of CP cases (**Figure 4**). The Tatton-Brown-Rahman syndrome, represented by one individual, was the only syndrome without an infection history. The most frequently described types of infections were respiratory tract infections (RTI, n=204/715, 28.5%), among which the most common were otitis media (OM, n=119/715, 16.6%) and pneumonia (PNA, n=67/715, 9.4%), followed by bronchiolitis (n=10/715, 1.4%) and sinusitis (n=6/715, 0.8%). RTI were not reported in very few syndromes, including Sotos syndrome, SBBYS syndrome, HSAIN1E/ADCA-DN syndrome, and Tatton-Brown-Rahman syndrome, which are, however, represented by only up to three cases, and hereditary sensory and autonomic neuropathy. Urinary tract infections (UTI) affected in total 2% of CP patients (n=14/715) with Sotos syndrome (90), SBBYS syndrome (136) Kabuki syndrome type 1 (77, 156–160) Weidemann-Steiner syndrome (64, 66), Kleefstra syndrome type 1 (58), Rubinstein-Taybi syndrome type 2 (121), ICF syndrome type 1 (147, 161) and MRAD 32 syndrome (131). Sepsis occurred in 15 individuals (2.1%) diagnosed with ICF syndrome type 1 (151, 152), Kabuki syndrome type 1 (85), Weidemann-Steiner syndrome (68, 69) and MRAD 32 syndrome (135). Dermal and bone infections affected 2.8% of CP patients (n=20/715), mainly with hereditary sensory and autonomic neuropathy (77% of cases), which in this case can be a result of self-injuries characteristic of the syndrome. Less frequently described infections included gastroenteritis and meningitis. Several other infections were reported once. Altogether, similar to the general population, infections of the respiratory tract are most common among CPs. Hereditary sensory and autonomic neuropathy was selectively associated with skin and bone infections (100, 101), Luscan-Lumish syndrome and ODLURO syndrome seem to be selectively associated with recurrent OM (87, 88, 104, 105) while SBBYS and Sotos syndrome are associated with UTI (90, 136). Yet for most of these syndromes, the data were available only for a few patients. Kabuki syndrome type 1, ICF syndrome type 1, and Rubinstein-Taybi syndrome patients present with a wide spectrum of infections, and sepsis was the most common among ICF syndrome type 1 (19% of cases) (**Figure 4**). These findings underline the variation in manifestation of infections across different CPs.

#### 3.2.2 Immunoglobulin levels

**Figure 5:**
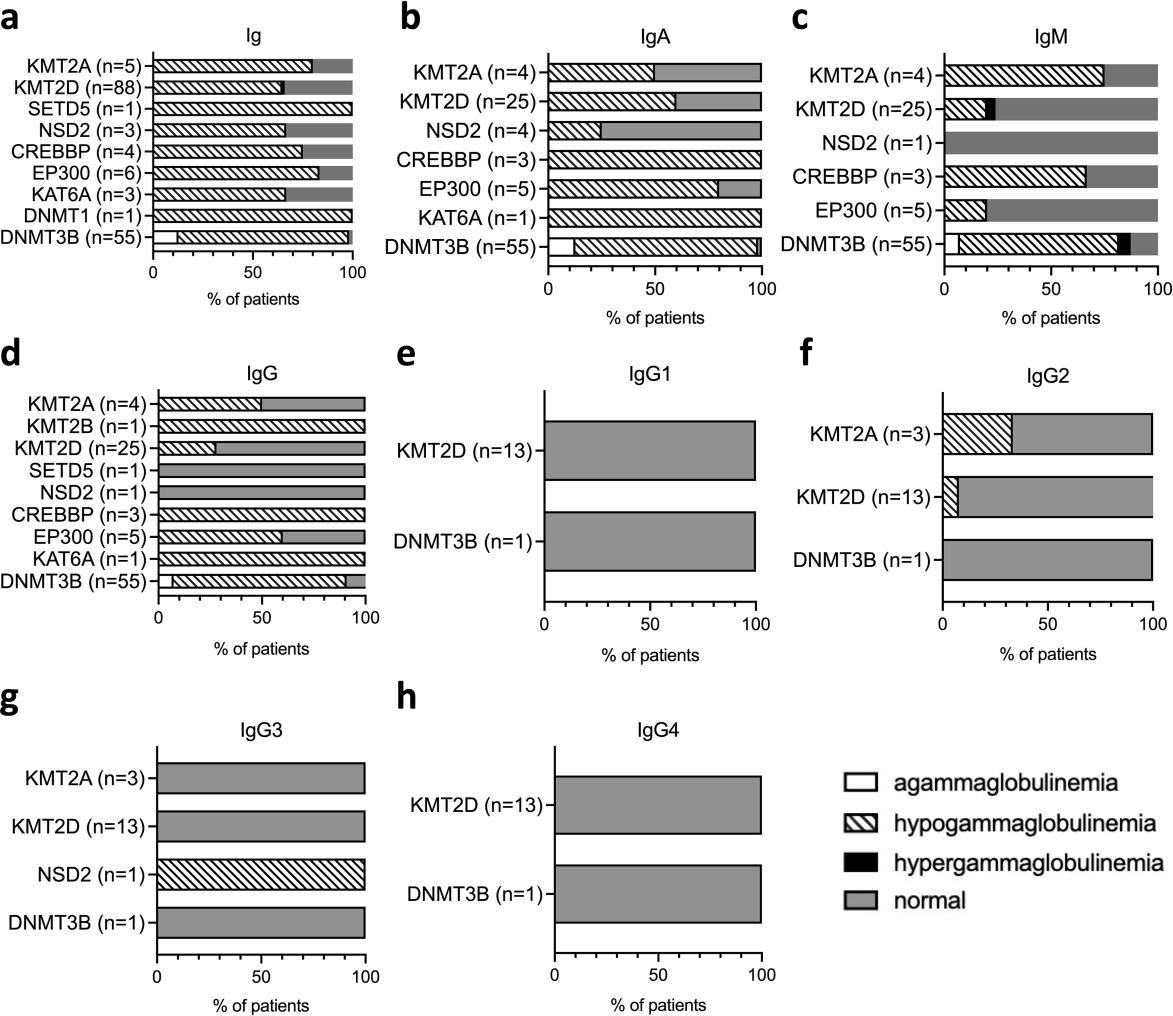
Immunoglobulin levels in individuals with different chromatinopathies resulting from mutations in epigenetic writers. Serum concentrations of **(a)** total immunoglobulin, **(b)** IgA, and **(c)** IgM as well as **(d)** total IgG and **(e–h)** its subclasses (IgG1–IgG4) are grouped as absent gammaglobulinemia (agammaglobulinemia), hypergammaglobulinemia, hypogammaglobulinemia, or normal in various chromatinopathies based on available reports. CREBBP, CREB-binding protein; DNMT, DNA methyltransferase; EP300, E1A-associated protein p300; Ig, immunoglobulin; KAT, lysine acetyltransferase; KMT, lysine methyltransferase; NSD, nuclear receptor-binding SET domain protein; SET, Su(var), Enhancer of zeste, Trithorax; SETD, SET domain-containing protein.

The underlying cause of increased susceptibility to infections in CPs could be a defect in immunoglobulin production. Immunoglobulin levels were available for 183 individuals diagnosed with ten different syndromes: Kabuki syndrome type 1 (n=101) (75–77, 79, 80, 83–86, 162), ICF syndrome type 1 (n=55) (141–148, 150–154, 161), Rubinstein-Taybi syndrome type 1 (n=4) (110, 111, 115, 116, 118, 119) and type 2 (n=6) (126–129), Wolf-Hirschhorn syndrome (n=6) (92, 94, 97, 98), Wiedemann-Steiner syndrome (n=5) (60, 63), MRAD 32 syndrome (n=3) (133), MRAD 23 syndrome (n=1) (107), HSAIN1E/ADCA-DN syndrome (n=1) (137), and DYT28 (n=1) (71) (**Figure 5**, **Supplementary Figure 2**). Only 23% of all CP individuals presented with normal total immunoglobulin levels (n=38/166; **Figure 5A**). The majority of the individuals presented with hypogammaglobulinemia (reduced immunoglobulin levels, n=120/166, 72%), and seven individuals (n=7/163, 4.3%), all diagnosed with ICF syndrome type 1, presented with agammaglobulinemia (very low concentrations of immunoglobulins). Among the syndromes most affected by hypogammaglobulinemia was ICF syndrome type 1, in which hypogammaglobulinemia regarded all Ig classes: IgA, IgM, and IgG (**Figures 5B–D**, **Supplementary Figure 2**). The most frequently reduced or absent immunoglobulin among CP patients was IgA (n=73/93, 78.5%; **Figure 5B**, **Supplementary Figure 2**). IgA levels were affected in all examined CP syndromes and were in the normal range in only 18.3% of cases. IgM and IgG levels were reduced or absent in more than half of the individuals (n=52/93, 56%, and n=63/96, 65.6%, respectively; **Figures 5C, D**, **Supplementary Figure 2**). ICF syndrome type 1, Wiedemann-Steiner syndrome, and Rubinstein-Taybi syndrome type 1 were the most affected by IgM and IgG deficiency. Compromised IgG levels were also found in MRAD 23 and HSAIN1E/ADCA-DN syndromes, which were, however, represented by only one individual. Regarding IgG subclasses, only IgG2 was affected in a few individuals with Wiedemann-Steiner syndrome or Kabuki syndrome type 1 (60, 76) and IgG3 levels in Wolf-Hirschhorn syndrome (98) (**Figures 5E–H**). Hypergammaglobulinemia affected one Kabuki syndrome type 1 patient and three ICF syndrome type 1 patients and regarded IgM (77, 151) (**Figure 5C**). Altogether, our systematic analysis revealed that antibody production is compromised in all ten syndromes, where IgA seems to be the most affected in all syndromes apart from Wiedemann-Steiner syndrome, where IgM levels are the most affected; DYT28 syndrome, where IgG deficiency was reported in one individual (71) and Wolf-Hirschhorn syndrome, where IgG3 deficiency was reported in one individual (98).

#### 3.2.3 Blood cell populations

**Figure 6:**
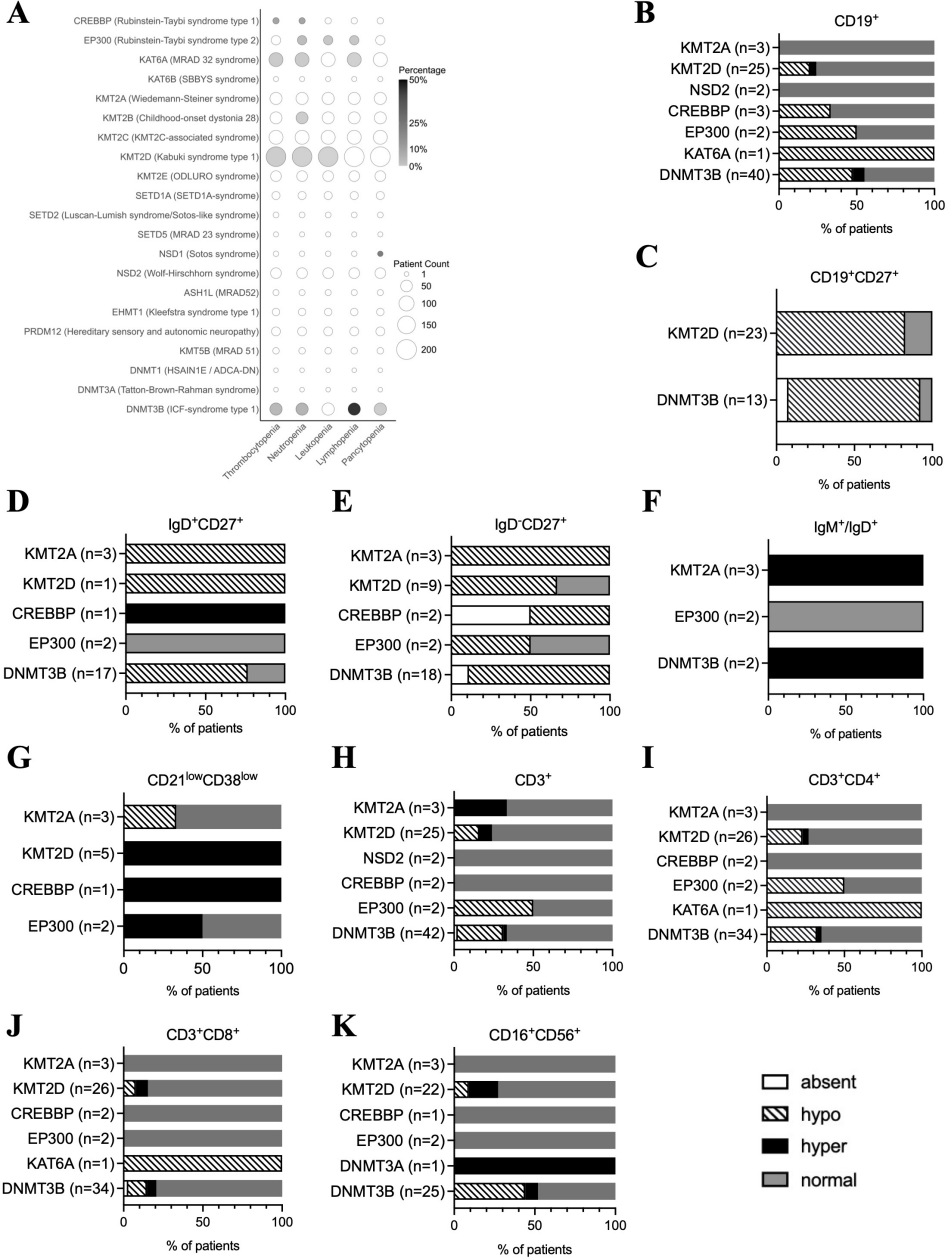
Peripheral blood immune cell populations in patients with different chromatinopathies. **(A)** Prevalence of cytopenias in blood, including thrombocytopenia, neutropenia, leukopenia, lymphopenia, and pancytopenia, reported in particular chromatinopathies. Frequencies of lymphocyte subsets obtained from flow cytometry analysis, including **(B)** total B cells, **(C)** memory B cells (CD19^+^CD27^+^), **(D)** unswitched memory B cells (IgD^+^CD27^+^), **(E)** switched memory B cells (IgD^+^CD27^+^), **(F)** naïve B cells (IgM^+^/IgD^+^), **(G)** CD21lowCD38low B cells, as well as **(H)** total T cells (CD3^+^), **(I)** CD4^+^ T cells, **(J)** CD8^+^ T cells, and **(K)** natural killer cells (CD16^+^CD56^+^), are classified as hyper, hypo, absent, or normal in different chromatinopathies based on available data. ADCA-DN, autosomal dominant cerebellar ataxia, deafness, and narcolepsy; ASH1L, absent, small, or homeotic-like 1; CREBBP, CREB-binding protein; DNMT, DNA methyltransferase; EHMT, euchromatic histone methyltransferase; EP300, E1A-associated protein p300; HSAIN1E, hereditary sensory and autonomic neuropathy 1E; ICF, immunodeficiency, centromeric region instability, facial anomalies; Ig, immunoglobulin; KAT, lysine acetyltransferase; KMT, lysine methyltransferase; MRAD, intellectual disability autosomal dominant; NSD, nuclear receptor-binding SET domain protein; PRDI-BF1, positive regulatory domain I binding factor 1; PRDM12, PRDI-BF1 and RIZ domain-containing protein; RIZ, retinoblastoma-interacting zinc-finger; SBBYS, Say-Barber/Biesecker/Young-Simpson syndrome; SET, Su(var), Enhancer of zeste, Trithorax; SETD, SET domain-containing protein.

In line with impaired immunoglobulin production, several blood cell defects, including dysregulation of B cell maturation, have been described in various CPs, especially in ICF and Kabuki syndromes (141, 163). In articles included in our review, lymphopenia was very common among ICF syndrome type 1 patients (**Figure 6A**). Neutropenia and thrombocytopenia were also mainly reported in ICF syndrome type 1 but also in Rubinstein-Taybi syndrome type 1 and type 2, Kabuki syndrome type 1, MRAD 32 syndrome, and DYT28 syndrome, while pancytopenia and leukopenia were reported once in Sotos syndrome, ICF syndrome type 1, Kabuki syndrome type 1, and Rubinstein-Taybi syndrome type 2 patients (71, 77, 79, 109, 110, 126–128, 148, 151, 154, 162) (**Figure 6A**). Blood B cell and T cell counts were available for 76 individuals, while NK cell counts were available for 54 individuals diagnosed with Kabuki syndrome type 1 (76, 77, 83, 86), Wiedemann-Steiner syndrome (60), Rubinstein-Taybi syndromes type 1 and type 2 (109–111, 115, 116, 119, 126–128), ICF syndrome type 1 (141–148, 151–154, 161), MRAD 32 syndrome (133), Tatton-Brown-Rahman syndrome (138) and Wolf-Hirschhorn syndrome (92, 94, 98) (**Figures 6B–K**). The frequency of circulating lymphocytes - total B (CD19^+^) cells, (CD3^+^) T cells, helper (CD4^+^) T cells, cytotoxic (CD8^+^) T cells, and total NK (CD16^+^56^+^) lymphocyte counts - was in the normal range in over half of all CP patients examined (**Figures 6B, H–K**). ICF syndrome type 1 was the most affected syndrome by abnormal B cell counts (**Figure 1B**). Interestingly, the majority of ICF syndrome type 1 and Kabuki syndrome type 1 patients, for whom the data was available, presented with reduced numbers or absent CD19^+^CD27^+^ memory B cells (n=31/36, 86%) (**Figure 6C**). Furthermore, IgD^-^CD27^+^ switched memory B cell counts seem to be more affected in CPs than IgD^+^CD27^+^ unswitched memory B cell counts (**Figures 6D, E**). Although the data on naïve IgM^+^/IgD^+^ B cell counts is limited to few patients, all ICF syndrome type 1 and Weidemann-Steiner syndrome patients show increased numbers of these cells (**Figure 6F**) (60, 126–128, 154, 161). This data collectively suggests a defect in B cell maturation in some CPs, such as ICF syndrome type 1, Kabuki syndrome type 1, and Wiedemann-Steiner syndrome. B cell deficiency was also observed in one of each: MRAD 32, Rubinstein-Taybi type 1, and type 2 syndrome patients, represented by only a few individuals (**Figure 6B**). The increased numbers of CD21^low^ B cells implicated in autoimmunity (164–167) were also reported in Kabuki syndrome type 1, Rubinstein-Taybi syndromes type 1 and type 2 (**Figure 6G**) (60, 77, 83, 115, 126–128) which in some cases were accompanied by autoimmune diseases (83, 115, 126–128). T cell frequencies were reduced or absent in almost one-fourth of CP patients (n=18/76, 24%) affected by Rubinstein-Taybi syndrome type 2, ICF syndrome type 1, and Wiedemann-Steiner syndrome, while 4 individuals had elevated T cell counts (n=4/76, 5.3%) (**Figure 6H**) (60, 77). CD4^+^ T cell numbers were more often reduced than CD8^+^ T cell numbers in these patients (n=18/68, 28%, and n=8/68, 12%, respectively) (**Figures 6I, J**). NK cell numbers were mostly in a normal range but were most altered in ICF syndrome type 1 and Kabuki syndrome type 1 patients, showing either increased or reduced frequencies in few patients (**Figure 6K**) (77, 138, 146, 151, 154, 161). One patient with Tatton-Brown-Rahman syndrome had lymphoproliferative disorder of these cells (138). Altogether this analysis underscores the dysregulation of the cellular compartment of peripheral blood in CP patients, with B cell populations and ICF syndrome type 1 being the most affected.

#### 3.2.4 Immunodeficiency

**Figure 7:**
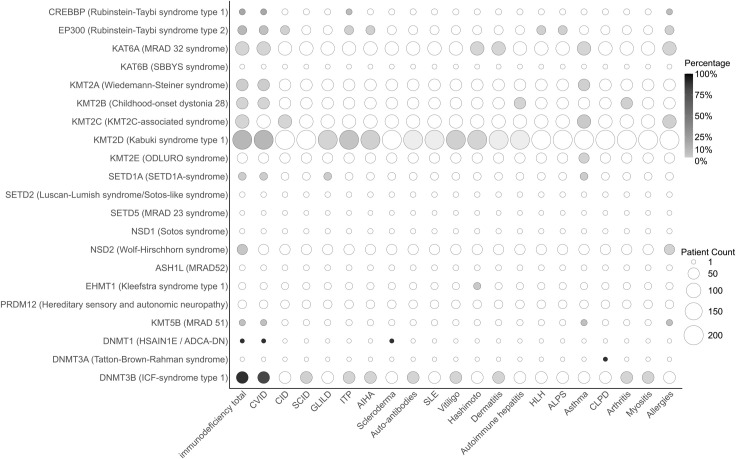
Prevalence of immunopathology across chromatinopathies resulting from mutation in epigenetic writers. The bubble plot depicts specific types of immune-related conditions on the x-axis in different chromatinopathies listed on the y-axis. The number of reported patients is illustrated as bubble sizes, whereas the percentage of incidences within each syndrome is displayed as bubble shading. ADCA-DN, autosomal dominant cerebellar ataxia, deafness, and narcolepsy; AIHA, autoimmune hemolytic anemia; ALPS, autoimmune lymphoproliferative syndrome; ASH1L, absent, small, or homeotic 1-like; CID, combined immunodeficiency disorder; CLPD, chronic lymphoproliferative disorder; CREBBP, CREB-binding protein; CVID, common variable immunodeficiency disorder; DNMT, DNA methyltransferase; EHMT, euchromatic histone methyltransferase; EP300, E1A-associated protein p300; GLILD, granulomatous and lymphocytic interstitial lung disease; HLH, hemophagocytic lymphohistiocytosis; HSAIN1E, hereditary sensory and autonomic neuropathy 1E; ICF, immunodeficiency, centromeric region instability, facial anomalies; ITP, immune thrombocytopenic purpura; KAT, lysine acetyltransferase; KMT, lysine methyltransferase; MRAD, intellectual disability autosomal dominant; NSD, nuclear receptor-binding SET domain protein; PRDI-BF1, positive regulatory domain I binding factor 1; PRDM12, PRDI-BF1 and RIZ domain-containing protein; RIZ, retinoblastoma-interacting zinc-finger; SBBYS, Say-Barber/Biesecker/Young-Simpson syndrome; SCID, severe combined immunodeficiency; SET, Su(var), Enhancer of zeste, Trithorax; SETD, SET domain-containing protein; SLE, Systemic lupus erythematosus.

Consistent with commonly found susceptibility to infections and hypogammaglobulinemia, immunodeficiency was commonly found and was diagnosed in 16.5% (n=118/715) of CP individuals (**Figure 7**). The most frequently described immunodeficiency was common variable immunodeficiency disorder (CVID, n=110/715, 15.4%), reported in most ICF syndrome type 1 (n=56/58, 97%) and Kabuki syndrome type 1 (n=40/197, 20%) (143, 145, 146, 153, 157, 159, 160), Rubinstein-Taybi syndrome type 1 (n=2/6, 25%) (116, 119) and type 2 (n=4/26, 15%) (126–128), Wiedemann-Steiner syndrome (n=3/60, 5%) (60) and single cases of SETD1A-syndrome (n=1/16, 6%) (102), MRAD 32 syndrome (n=1/83, 1%) (133, 137), HSAIN1E/ADCA-DN syndrome (n=1/1, 100%) (137) and MRAD 51 syndrome (n=1/7, 14.3%) (89). Few of the individuals with Kabuki syndrome type 1 and one individual with SETD1A syndrome affected by CVID also presented with granulomatous and lymphocytic interstitial lung disease (GLILD) (n=5/197, 3%, and n=1/16, 6%, respectively) (75, 102, 159, 160) which is a life-threatening pulmonary complication of CVID (81). Furthermore, two cases of combined immunodeficiency disorder (CID) in KMT2C-related syndrome (73) and Rubinstein-Taybi syndrome type 2 (126–128) and two severe CID (SCID) in ICF syndrome type 1 (148) were also described. Altogether, immunodeficiency was found in eleven of the analyzed CP syndromes, with the highest prevalence in ICF syndrome type 1.

#### 3.2.5 Autoimmunity

Autoimmunity is another common manifestation of immune dysregulation within CPs (**Figure 7**). The most frequently described autoimmune manifestation was immune thrombocytopenic purpura (ITP, n=25/715, 3.5%) which affected 11% of the individuals with Kabuki syndrome (22/197) (77, 79, 82–84, 86, 156, 158, 168–171) as well as some patients with ICF syndrome type 1 (146, 152, 154) and Rubinstein-Taybi syndromes (115, 116, 126–128). As a comparison, the annual incidence of ITP among pediatric patients is estimated to be 1-13.4 cases per 100,000 people (172–174). Less frequently observed were autoimmune hemolytic anemia (AIHA, n=10/715, 1.4%) in Rubinstein-Taybi syndrome type 2 (126–128), Kabuki syndrome type 1 (79, 83, 85, 86) and ICF syndrome type 1 (154); vitiligo (n=9/715, 1.3%) in Kabuki syndrome type 1 (78, 85) and ICF syndrome type 1 (142, 154). The frequency of vitiligo among CPs is higher than the estimated prevalence of 0.5-1% globally (175). Hashimoto thyroiditis was reported in 1% of CP patients (n=7/715) with Kabuki syndrome type 1 (78), KMT2C-associated syndrome (56), and MRAD32 syndrome (133). The prevalence of pediatric thyroiditis in the US is estimated to be 3.7% (176), while the overall prevalence in adults is 7.5% (177) indicating that CP patients do not have increased susceptibility to developing Hashimoto disease. Other reported autoimmune manifestations included hemophagocytic lymphohistiocytosis (HLH) and autoimmune lymphoproliferative syndrome (ALPS) (126–128), myositis (151), arthritis (71, 151), autoimmune hepatitis (71, 77), and systemic lupus erythematosus (SLE) (80). Three patients with KMT2C deficiency were also reported with autoimmune/allergic features but without specifying the disease (80). Among the syndromes most affected by autoimmunity were Kabuki syndrome type 1, Rubinstein-Taybi syndrome type 2, and ICF syndrome type 1.

#### 3.2.6 Other immunopathology

Among other immunopathologies, asthma affected 1.7% of CP individuals (n=12/715) with KMT2C-associated syndrome (n=6/82, 7%) (74), Wiedemann-Steiner syndrome (n=2/60, 3%) (68), KMT2E deficiency (n=1/39, 3%) (87), SETD1-syndrome (n=1/16, 6%) (103) and MRAD 51 syndrome (n=1/7, 14.2%) (89). Nine individuals among MRAD 32 syndrome (133), MRAD 51 syndrome (89), KMT2C-associated syndrome (74), Wolf-Hirschhorn syndrome (98) and Rubinstein-Taybi syndrome type 1 and type 2 presented with various allergies (117, 126–128) (**Figure 7**). No immunopathology was reported for patients with MRAD 52 syndrome, ODLURO syndrome, Luscan-Lumish syndrome, and SBBYS syndrome, which were, however, represented by very few cases, as well as hereditary sensory and autonomic neuropathy. These findings highlight the high occurrence of immunopathology in CPs with high variability in immunopathological presentations.

## 4 Discussion

Our systematic analysis revealed increased susceptibility to infections or immunopathology across all CP syndromes analyzed, often accompanied by dysregulated immunoglobulin production and abnormal lymphocyte development. Three of the CP syndromes included in this review: Wiedemann-Steiner syndrome, Kabuki syndrome type 1, and ICF syndrome type 1 have already been classified as disorders with inborn errors of immunity: monogenic disorders of the immune system (178). Consistently, we found these syndromes to be most affected by immunopathology. Our systematic review reveals immune dysregulation in other CPs such as Rubinstein-Taybi syndrome, MRAD 32 syndrome, DYT 28, Kleefstra syndrome type 1, KMT2C-associated syndrome, and Wolf-Hirschhorn syndrome, as well as points to the importance of a better evaluation of immune parameters within less characterized SETD1A syndrome, HSAIN1E/ADCA-DN, Sotos syndrome, Tatton-Brown-Rahman syndrome, SBBYS syndrome, MRAD 23 syndrome, MRAD 51 and 25 syndromes, hereditary sensory and autonomic neuropathy, and Luscan-Lumish syndrome.

### 4.1 Germline mutations in histone methyltransferases and resulting immunopathology

Chromatinopathies resulting from defects in histone methyltransferases consist of the biggest group of patients in our review, in line with the big number of enzymes catalyzing the deposition of methyl groups on different residues of histone tails (179). The most represented syndrome in our review is Kabuki syndrome type 1. The cause of the syndrome is a mutation in the *KMT2D* gene in up to 90% of cases (180, 181), whereas 5% of the patients have a mutation in the *KDM6A* gene, which encodes a histone demethylase; these cases are often referred to as Kabuki syndrome type 2 (182). Because KDM6A belongs to the “eraser” category of epigenetic enzymes, our review did not include individuals with KDM6A mutations (3). We also did not include individuals without a genetically confirmed Kabuki syndrome diagnosis due to the uncertainty of the condition, although many reports on the immunological status of clinically diagnosed patients exist (156–160, 168–171, 183–188). Consistent with molecularly diagnosed KMT2D mutations, patients with a clinical diagnosis of Kabuki syndrome showed susceptibility to infections, especially RTIs (75, 156–160, 169–171, 185, 187, 188), as well as a broad range of other infections (156–160, 184–188). According to previous reports, hypogammaglobulinemia affected most of the patients (156–160, 162, 169, 170, 185, 188) similar to our study. In both clinically and molecularly diagnosed patients, it mostly affected the IgA isotype (76, 77, 83, 86, 156–160, 169, 170, 185). In both cases, most Kabuki syndrome patients had normal T and B cell counts, with few cases of increased B cell and T cell numbers (77, 84). However, they consistently showed reduced memory B cell numbers and elevated naïve and CD21^low^CD38^low^ B cell numbers (76, 77, 83, 86, 159, 160). Yet, in Kabuki syndrome type 1 patients with molecular diagnosis, CVID was twice as common (20%) as in clinically diagnosed patients (8%) (85, 157). Regarding autoimmune and inflammatory manifestations, ITP (77, 79, 82–86, 156, 158, 162, 168–170), AIHA (79, 83, 85, 86, 162, 169), GLILD (81, 85, 159, 160), vitiligo (77, 78, 85, 169, 186, 188), and autoimmune thyroiditis (78, 85, 186) were reported in both groups of patients. However, auto-antibodies (158), Henoch-Schönlein purpura (HSP) (187), hyper IgM syndrome, and organizing pneumonia (183) were found only among clinically diagnosed Kabuki syndrome patients. Altogether our analysis demonstrates immunodeficiency and autoimmunity being common among Kabuki syndrome patients independently of the molecular diagnosis. Furthermore, mouse models have reported a critical role of Kmt2d in lymphocyte development (180, 189). Current mouse models partially recapitulated the T cell phenotype observed in patients (190), and a defective regulatory T cell development upon Kmt2d deficiency in mice might partially explain the high prevalence of autoimmunity in Kabuki syndrome patients (191).

Another well-represented syndrome in our analysis caused by mutations in H3K4 methyltransferase is Wiedemann-Steiner syndrome (62, 192). Patients with *KMT2A* mutations were sometimes classified as atypical Kabuki or Rubinstein-Taybi–like syndrome, which highlights the shared biological pathways among KMT2A, KMT2D, CREBBP, and EP300 (193, 194). Yet, according to our analysis, the prevalence of infections and immune-related diseases is lower in Wiedemann-Steiner syndrome than in Kabuki and Rubinstein-Taybi syndromes. RTI and UTI affected 22% and 5% of Wiedemann-Steiner syndrome patients, respectively, and in contrast to Rubinstein-Taybi and Kabuki syndromes, other infections were not reported among Wiedemann-Steiner syndrome patients apart from 2 cases of sepsis. A similar frequency of infections was found in a retrospective, multicenter, observational study of 104 individuals with molecularly confirmed Wiedemann-Steiner syndrome (195). The study also reported abnormal immunoglobulin levels in 53% and impaired vaccine responses in 31% of Wiedemann-Steiner syndrome patients. In the current study, hypogammaglobulinemia occurred in 4 out of 5 patients, predominantly affecting IgM. Yet, the CVID was diagnosed in 5% of cases, and no additional immunopathology was observed, aside from two asthma cases (68). Although Kmt2a has been shown to be important for hematopoiesis in mice (196–199), our review did not reveal abnormal blood cell counts. Wiedemann-Steiner syndrome patients also had normal numbers of B cells but increased naïve and reduced memory B cells. The T cell compartment was less affected. Consistently, conditional Kmt2a deletion suggests a predominant role in B cell homeostasis, though late CD4^+^ T cell differentiation is also impaired (200, 201). Altogether this data suggests the need for more immunological examinations of Wiedemann-Steiner syndrome patients.

Other enzymes catalyzing H3K4 methylation within the COMPASS complex, KMT2B and KMT2C, were found to be mutated among individuals included in our review. Reported immunopathology among these patients included asthma, allergies, and single cases of RTI, IgG deficiency, CVID accompanied by autoimmune hepatitis and multiarticular juvenile arthritis as well as CID, and recurrent infections (70–74, 202, 203). KMT2E, another member of the KMT2 family, may lack intrinsic methyltransferase activity despite a conserved SET domain, potentially influencing H3K4 methylation indirectly. KMT2E-related neurodevelopmental disorder (ODLURO) has been recently defined, with only a few cases reported to date, in which recurrent respiratory infections and asthma were the only immunopathologies observed (74, 88, 204–206). SETD1A-syndrome is another only recently characterized disease caused by the mutation in H3K4 methyltransferase SETD1 (102). Recurrent RTIs, asthma, and CVID accompanied by GLIDL were reported among 16 of these patients (102, 103). Altogether, mutations in H3K4 methyltransferases may increase the susceptibility to infections and often result in immunodeficiency and other immunopathology.

CPs resulting from mutations in histone H3K36 methyltransferases constitute another group of patients reported in the review. Our analysis included a total of 53 patients with NSD1, NSD2, SETD2, SETD5, and ASH1L mutations, with Wolf-Hirschhorn syndrome being the most represented. Its pathology is multigenic, with severity determined by the deletion size of a short arm of chromosome 4 (4p16.3) containing NSD2 (93, 207). Early reports found a high prevalence of immunoglobulin deficiency (9/13), mainly IgA, respiratory and viral infections, CVID in some cases, and normal T cell immunity among the Wolf-Hirschhorn syndrome patients, but these early reports lack detailed genetic information (208, 209). In genetically confirmed NSD2 cases, respiratory infections and immunodeficiency, including IgA and IgG3 hypogammaglobulinemia, were observed, but immunoglobulin data were limited (≤4 patients per subtype) (92, 97–99). Two patients had normal lymphocyte counts (92, 94, 98). Yet, mouse models implicate NSD2 in CD4^+^ T cell subsets and B cell development, highlighting the need for further immunological evaluation (210–213). Mutations in NSD1 and SETD2 cause Sotos syndrome and Luscan–Lumish syndrome/Sotos-like syndrome, respectively, which resemble each other and are characterized by overgrowth, macrocephaly, similar facial features, and learning disability (90, 91, 105). While OM was reported in most Luscan–Lumish syndrome/Sotos-like syndrome patients (104, 105), UTI (90) or pancytopenia (91) were reported in Sotos syndrome patients. A recent study revealed impaired B cell development, germinal center formation, and antibody production by NSD1-deficient B cells in mice (214). Similarly, SETD2 has been shown to be important for hematopoiesis (215), V-D-J recombination in B cells and T cells (216), and for controlling autoimmunity and inflammatory responses in mice (216–220). Altogether these findings highlight the importance of further immunological evaluation of Sotos and Soto-like syndrome patients. Mutations in the *ASH1L* gene have been associated with MRAD 52 syndrome just recently, with approx. 60 cases described in the clinic (221). Our search identified 5 patients, one with an infection history (89). Similarly, although MRAD 23 syndrome was represented by only two cases, both had RTI, and one had reduced IgG levels (106, 107) suggesting an immunodeficiency. The last two syndromes from the histone methyltransferase group in our review are Kleefstra syndrome type 1 and hereditary sensory and autonomic neuropathy caused by mutations in H3K9 methyltransferases: EHMT1 and PRDM12, respectively. While a broad range of infections were reported among Kleefstra syndrome type 1 patients (74), hereditary sensory and autonomic neuropathy was associated specifically with dermal and bone infections (100, 101). Yet, these infections are most likely the consequence of persistent self-injuries, rather than developmental immune defects, as PRDM12 mutations manifest in congenital insensitivity to pain.

### 4.2 Germline mutations in histone acetyltransferases and resulting immunopathology

Histone acetyltransferases consist of a big group of proteins (222), yet mutations in four of them—CREBBP, EP300, KAT6A, and KAT6B—have been associated with congenital disorders so far. Germline mutations in CREBBP account for approx. 55% of Rubinstein-Taybi syndrome patients and in EP300 for approx. 8%, categorized as type 1 and type 2, respectively (120, 223). The proteins are highly homologous transcriptional coactivators, yet they regulate distinct biological pathways (224–228). They have been involved in hematopoiesis as well as peripheral B cell and T cell development (229–237). Rubinstein-Taybi syndrome was first described in seven similar cases of the congenital disorder characterized by short, broad thumbs and great toes as well as psychomotor retardation, highly arched palates, and particular facial abnormalities (238). RTIs were noted in all seven children in the original description of the syndrome, with five also presenting allergy-related conditions (eczema, hay fever, asthma, and food allergies). Subsequent reports have consistently described recurrent infections in additional cases (239–242). For most of these cases, the syndrome was diagnosed based on clinical parameters, and the genetic analysis was not available. Our search strategy identified infection history and clinical data from 12 genetically confirmed Rubinstein-Taybi syndrome type 1 patients and 26 Rubinstein-Taybi syndrome type 2 patients (108–119, 121–130). Consistent with previous reports, the prevalence of infections was high among the patients. Most Rubinstein-Taybi syndrome patients had a history of RTIs. Other infections affecting Rubinstein-Taybi syndrome included gastroenteritis, esophagitis, bulbitis, gastritis, dermal infections, meningitis, and sepsis (241). As recurrent respiratory infections in many cases might result from microaspiration or gastro-oesophageal reflux (239), they have also been suggested to be associated with a commonly diagnosed hypogammaglobulinemia (110, 116). In our study, immunoglobulin deficiency was found in most Rubinstein-Taybi syndrome patients, where the most affected immunoglobulin isotypes were IgA and IgG. In a very comprehensive immunological evaluation of 97 patients with Rubinstein-Taybi syndrome (some cases included in our study), the authors found hypogammaglobulinemia, mainly affecting IgA and IgM levels, to be the most frequent immune manifestation, affecting one-fifth of the patients (241). Furthermore, authors reported increased IgM levels in 30% of their cohort, while our study found only one Rubinstein-Taybi syndrome type 1 patient to have IgM hypergammaglobulinemia. The discrepancy in prevalence between the studies could be due to a different genetic background of the disease in analyzed patients and probably to our research strategy that could be biased towards patients manifesting with a more severe immunological phenotype in contrast to a general population of Rubinstein-Taybi syndrome patients described by Saettini et al. Unfortunately, these records could not be included in our study, as the results specific to Rubinstein-Taybi syndrome type 1 and type 2 patients could not be retrieved. Interestingly, apart from a general immunoglobulin deficiency, specific defects of pneumococcal antibody production seem to be common among these patients, reflecting difficulties with responses to pneumococcal polysaccharide antigens (116, 241). Furthermore, the authors observed lymphopenia in 8.3% of patients (241) while in our study few patients presented with leukopenia, lymphopenia, neutropenia, or thrombocytopenia, and few were diagnosed with CVID or CID. Interestingly, two Rubinstein-Taybi syndrome type 1 patients with leukocytosis even in the absence of infection were also reported (112, 113, 117). One of these patients had a defect in granulopoiesis affecting half of the blood neutrophils (112, 113, 241). Furthermore, we observed reduced counts of memory B cells in the majority of patients, in particular switched memory B cells, which was consistent with the observation by Saettini et al. (241). One Rubinstein-Taybi syndrome type 2 patient included in our and Saettini et al. study had a clinical profile resembling autoimmune lymphoproliferative syndrome (ALPS), hemophagocytic lymphohistiocytosis (HLH), and autoimmune hemolytic anemia (AIHA) with expanded CD21^low^CD38^low^ B cells (126–128, 241). Furthermore, our study identified Rubinstein-Taybi syndrome type 1 with the expansion of this B cell population, which was accompanied by ITP and neutropenia (115). In the previous report, autoimmune or autoinflammatory manifestations were observed in 12.3% of cases and lymphoproliferation in 8.2% of patients (241). Taken together, these findings suggest immune defects in Rubinstein-Taybi syndrome mainly manifesting in increased susceptibility to infections, hypogammaglobulinemia, and a defect in the generation of memory B cells.

Other acetyltransferases mutated in CPs include KAT6A, which causes autosomal dominant intellectual disability (MRAD32; also known as Arboleda–Tham syndrome) (131, 243, 244), and KAT6B, initially linked to Say–Barber/Biesecker/Young–Simpson syndrome (SBBYS; also known as Ohdo (245) and genitopatellar syndromes) (246, 247), and more recently associated with a wide range of congenital disorders (133, 247–251). The proteins are structurally related (252, 253) and both have been shown to regulate immune processes in mice (254–261). While RTIs were common among MRAD32 syndrome patients, they were not reported in the two patients with KAT6B mutations in our study. Instead, one of the two patients reported UTIs (136). Other infections sporadically reported in patients with KAT6A mutation included conjunctivitis, sepsis, herpes simplex infections of the face and eyes causing keratitis, impetigo, and *Staphylococcus* infections (133). In two cases, recurrent infections were reported together with blood results such as neutropenia with low immunoglobulin levels, leading to the CVID diagnosis or lymphopenia. One KAT6A patient presented with severe intermittent neutropenia but without recurrent infections (132, 133). Other immune disorders reported among KAT6A-related syndrome patients included thrombocytopenia, Hashimoto disease, and eczema (132, 133). Although there is currently no link established between susceptibility to infections and immune dysregulation in these patients, the laboratory results were not available for most of the patients. Altogether, among CPs caused by mutations in histone acetyltransferases, Rubinstein-Taybi syndromes have the highest prevalence of immune-related phenotypes manifesting in increased susceptibility to infections, most likely due to the maturation defect of B cells.

### 4.3 Germline mutations in DNA methyltransferases and resulting immunopathology

Three enzymes—DNMT1, DNMT3A, and DNMT3B—directly catalyze the addition of methyl groups onto DNA. Whereas somatic mutations of DNMTs are very often found in clonal hematopoiesis and leukemias, germline mutations in each of the enzymes lead to neurological multisystem disorders (262). Germline mutations of DNMT1 cause HSAIN1E and ADCA-DN syndromes, which are primarily degenerative disorders of the central and peripheral nervous systems (263–265). DNMT1 is highly expressed in immune cells and has been shown to play a role during hematopoiesis (266) and lymphocyte development (267, 268). Our search strategy resulted in the identification of one patient with overlapping HSAIN1E and ADCA-DN syndromes who was diagnosed with CVID on the basis of repeated infections and low immunoglobulin levels, scleroderma spectrum disorder, and encephalitis in addition to neurological problems (137). The inflammatory and immunodeficient phenotypes may reflect DNMT1 involvement in T cell homeostasis (267) and in B cell responses (268).

Although closely related in structure, DNMT3A and DNMT3B exhibit distinct expression patterns as well as different substrate and target DNA site preferences (269). Consistently, germline mutations in the DNMT3A and DNMT3B genes result in two distinct syndromes: Tatton-Brown-Rahman syndrome and ICF syndrome type 1, respectively. Although immune dysregulation is not very pronounced in Tatton-Brown-Rahman syndrome, severe immunodeficiency and hypogammaglobulinemia are a hallmark of ICF syndrome type 1, which causes the patients to succumb to infectious diseases before adulthood (141, 146, 150, 151, 153, 270). The recurrent infections encompass a broad range, but the most commonly reported include RTIs (pneumonia and otitis media), sepsis, gastroenteritis, and meningitis. Hypogammaglobulinemia, or absence of Igs, was present in all ICF syndrome type 1 reported cases. It may involve reduction of IgG, IgG subclasses, IgA, and/or IgM levels (146, 151, 153) and might evolve over time. For example, one individual with ICF syndrome type 1 demonstrated an Ig isotype switch in hypogammaglobulinemia, first presenting as reduced IgA and IgG in early childhood, followed by normalization of IgG and reduction of IgA and IgM later in life (150). Another patient had elevated IgM levels at age 26, which later declined to normal by age 33 (151). Half of ICF syndrome type 1 patients exhibit abnormal B lymphocyte counts, particularly reduced memory B cells, while naïve B cell numbers are increased, suggesting a defect in memory B cell formation (151). Although less described than B cell pathology, T cell and NK cell defects can also be found in ICF syndrome type 1 (271). Our analysis revealed a reduction in T cell counts that affected CD4^+^ and CD8^+^ T cell populations in almost half of patients, as well as a reduction or increase in NK cell numbers. Indeed, recent detailed evaluations of T cell populations in ICF syndrome type 1 patients revealed T cell abnormalities, including inverted CD4^+^/CD8^+^ T cell ratios, decreased counts of recent thymic emigrant cells (RTEs), dysbalance between naïve and memory T cell frequencies, elevated numbers of follicular T helper cells, and reduced numbers of regulatory T cells. Furthermore, their activation and functional profiles were also affected compared to healthy controls (145, 151, 154). Reduced lymphocyte proliferation in response to antigenic stimulation, as reported in some patients, along with impaired T cell maturation in the thymus, may partially explain the lymphocyte defects (151, 154). Additionally, several studies have reported inflammatory manifestations in ICF syndrome type 1 patients (272–274). Chromosomal instability, dysregulated expression of numerous genes involved in immune system function, and altered histone modification profiles have been observed in lymphoblastoid cells derived from patient material (275). Interestingly, leukocytes appear to be one of the most sensitive cell types to DNMT3B deficiency, pointing to an important role of the enzyme in leukocyte development.

Tatton-Brown-Rahman syndrome, first described in 2014 (263), has one reported case of immune dysregulation with a chronic NK-cell lymphoproliferative disorder in our review (138). Single-cell RNA sequencing of blood cells in another patient confirmed NK cell expansion, together with the increase in NK T cell numbers, reduction in naïve CD4^+^ T cell numbers, and broader transcriptional alterations in CD4^+^, CD8^+^, and monocyte subsets (276). This indicates that immune dysregulation in Tatton-Brown-Rahman syndrome might be more pronounced than reported and might require further monitoring. Altogether, among the three syndromes resulting from a deficiency in DNA methylation, the ICF syndrome manifests with the most severe immunopathology.

### 4.4 Phenotypic variability

The same epigenetic mutation does not necessarily result in the same immunophenotype. For example, two individuals with identical mutations in *EP300* presented with different immunological manifestations (127). One patient exhibited reduced IgA levels and a defect in the B and T cell compartment, resulting in increased susceptibility to infections, while the other patient had reduced IgA levels but normal lymphocyte counts and no history of recurrent infections. Furthermore, monozygotic twin boys affected by Wiedemann-Steiner syndrome were described, presenting with recurrent and severe infections and pan-hypogammaglobulinemia (60). Contrastingly, their mother, who also has Wiedemann-Steiner syndrome, presented with recurrent infections but had reduced levels of only IgM and reduced numbers of switched memory B cells (60). Moreover, one individual with Wiedemann-Steiner syndrome presented with cytomegalovirus and recurrent infections despite normal Ig levels (63). This is not in line with previously reported individuals presenting with hypogammaglobulinemia (60). These findings underline the clinical heterogeneity in immunopathology in CPs.

### 4.5 Limitations

This study has several limitations. Unequal syndrome representation might have skewed results. Thirty individuals were reported multiple times (duplicate, n=29; triplicate, n=1) (93–98, 109, 110, 126–128, 159, 160, 277). In two cohort studies, some individuals were reported in both, without specifying which one was reported twice (156, 169), resulting in inclusion uncertainty. One report on 23 individuals, including three previously described cases, provided infection history for the entire cohort without specifying individual patients, thereby preventing assignment of symptoms to duplicated cases (146). Some articles reported normal immunological values without actual numerical data, reporting only percentages of lymphocyte subpopulations rather than absolute counts, which precluded comparison. Furthermore, in some cases only a note on normal blood cell counts or lymphocyte populations was provided without further specification. Discrepancies existed between some conclusions of articles and reference values. Reporting of infection history varied between studies, among which many did not specify the infections (53, 55, 74, 82, 85, 86, 106, 133, 137, 144). If infections were not reported, it was assumed that there were none, possibly underestimating infection incidence among individuals with CP, especially since respiratory infections are common in children and therefore might not have been documented in their medical history. Finally, the search strategy could not include all available research articles. For example, 41 articles were added by cross-referencing; however, some could have been missed.

## 5 Conclusions

Our systematic analysis revealed infection history, especially recurrent RTI, and common hypogammaglobulinemia in various CPs, and B cell maturation defects in some patients consistent with previous reports. It remains to be established whether susceptibility to infections is a common feature of CPs caused by mutations in epigenes from the “writer” category, as expert opinions suggest that 6–10 self-limiting viral infections per year is within the normal range for healthy children (278, 279). As in some cases hypogammaglobulinemia can explain the recurrent infections in the CP patients and can even be linked to the defect in B cell maturation, but in most of the cases the underlying cause of immunopathology is unknown. Yet, the high prevalence of hypogammaglobulinemia emphasizes the existence of (latent) immune defects among CP patients. Impaired B cell maturation and immunoglobulin production observed in CP patients points to the importance of epigenetic machinery for the proper development of these cells and requires further investigation.

Interestingly, chromatinopathies present with an unusual breadth of the clinical phenotypes (4). Consistently, CP patients with the same syndrome show marked variability in immunological manifestations, possibly reflecting partial rather than complete loss of protein function or distinct mutation-specific effects on protein activity. Finally, since epigenetic mechanisms bridge the environmental influences with genetic predispositions, the wide spectrum of the phenotype in each syndrome could be the result of the influence of environmental exposures. Given the ambiguities in the inclusion of individuals in this analysis, variability in the documentation of immunological parameters, and discrepancies in reported infection histories, cautious interpretation of the data is warranted. With regard to future studies, standardized reporting guidelines for immunological laboratory data and larger cohort studies would enable more reliable analysis and better understanding of immune dysfunction in CPs, providing valuable insights for future research studies and clinical management strategies. It is therefore recommended to introduce immunoglobulin measurement in CP patients as a standard examination, especially in case of a history of recurrent, chronic, or severe infections and in less described syndromes. The assessment of blood immune cell populations would also allow better understanding of the immune status of patients.

## Data Availability

The original contributions presented in the study are included in the article/**Supplementary Material**. Further inquiries can be directed to the corresponding authors.

## Glossary

ADCA-DN: autosomal dominant cerebellar ataxia, deafness, and narcolepsy
AIHA: autoimmune hemolytic anemia
ALPS: autoimmune lymphoproliferative syndrome
ASH1L: absent, small, or homeotic 1-like
BCS: brittle cornea syndrome
CD: cluster of differentiation
CID: combined immunodeficiency disorder
CLP: common lymphoid progenitor
CMP: common myeloid progenitor
COMPASS: complex of proteins associated with Set1
CP: chromatinopathy
CREBBP: cAMP response element-binding protein
CVID: common variable immunodeficiency disorder
DN: double negative
DNA: deoxyribonucleic acid
DNMT: DNA methyltransferase
DP: double positive
DYT28: dystonia 28
EHMT: euchromatic histone methyltransferase
EP300: E1A-associated protein p300
EZH2: enhancer of zeste 2 polycomb repressive complex 2 subunit
FOXP3: forkhead box p3
GLILD: granulomatous and lymphocytic interstitial lung disease
H: histone
HLH: hemophagocytic lymphohistiocytosis
HSAIN1E: hereditary sensory and autonomic neuropathy 1E
HSC: hematopoietic stem cells
HSP: Henoch-Schönlein purpura
ICF: immunodeficiency, centromeric region instability, facial anomalies
Ig: immunoglobulin
ITP: immune thrombocytopenic purpura
K: lysine
KAT: histone acetyltransferase
KMT: histone methyltransferase
MDEM: mendelian disorder of the epigenetic machinery
me: methylation
mNDD: monogenetic neurodevelopmental disorder
MRAD: intellectual disability autosomal dominant
NF-kB: nuclear factor ‘kappa-light-chain-enhancer’
NK: natural killer
NSD: nuclear receptor-binding SET domain protein
ODLURO: O’Donnell-Luria-Rodan syndrome
OM: otitis media
PID: primary immunodeficiency
PNA: pneumonia
PRDI-BF1: positive regulatory domain I-binding factor 1
PRDM: PRDI-BF1 and RIZ domain-containing protein
PRISMA: preferred reporting items for systematic reviews and meta-analysis
RIZ: retinoblastoma protein-interacting zinc finger gene 1
RNA: ribonucleic acid
RTI: respiratory tract infection
SBBYS: Say-Barber/Biesecker/Young-Simpson syndrome
SCID: severe combined immunodeficiency disorder
SET: suppressor of variegation, enhancer of zeste, trithorax
SETD: SET domain-containing protein
SMYD2: SET and myeloid-nervy-DEAF1 domain containing 2
SP: single positive
Th: helper T
TREC: T receptor excision circle
Treg: regulatory T
UTI: urinary tract infection
VDJ: variable-diversity-joining.
