# Supplementary material for: Clinical immunology in chromatinopathies: a scoping review
Source: Front Immunol. 2026 Mar 26;17:1773284. doi: 10.3389/fimmu.2026.1773284 (PMC13062231; doi:10.3389/fimmu.2026.1773284)
Supplement: Supplementary file 1 [file DataSheet1.docx]

Supplementary Material

# Supplementary Figures and Tables

## Supplementary Figures

**
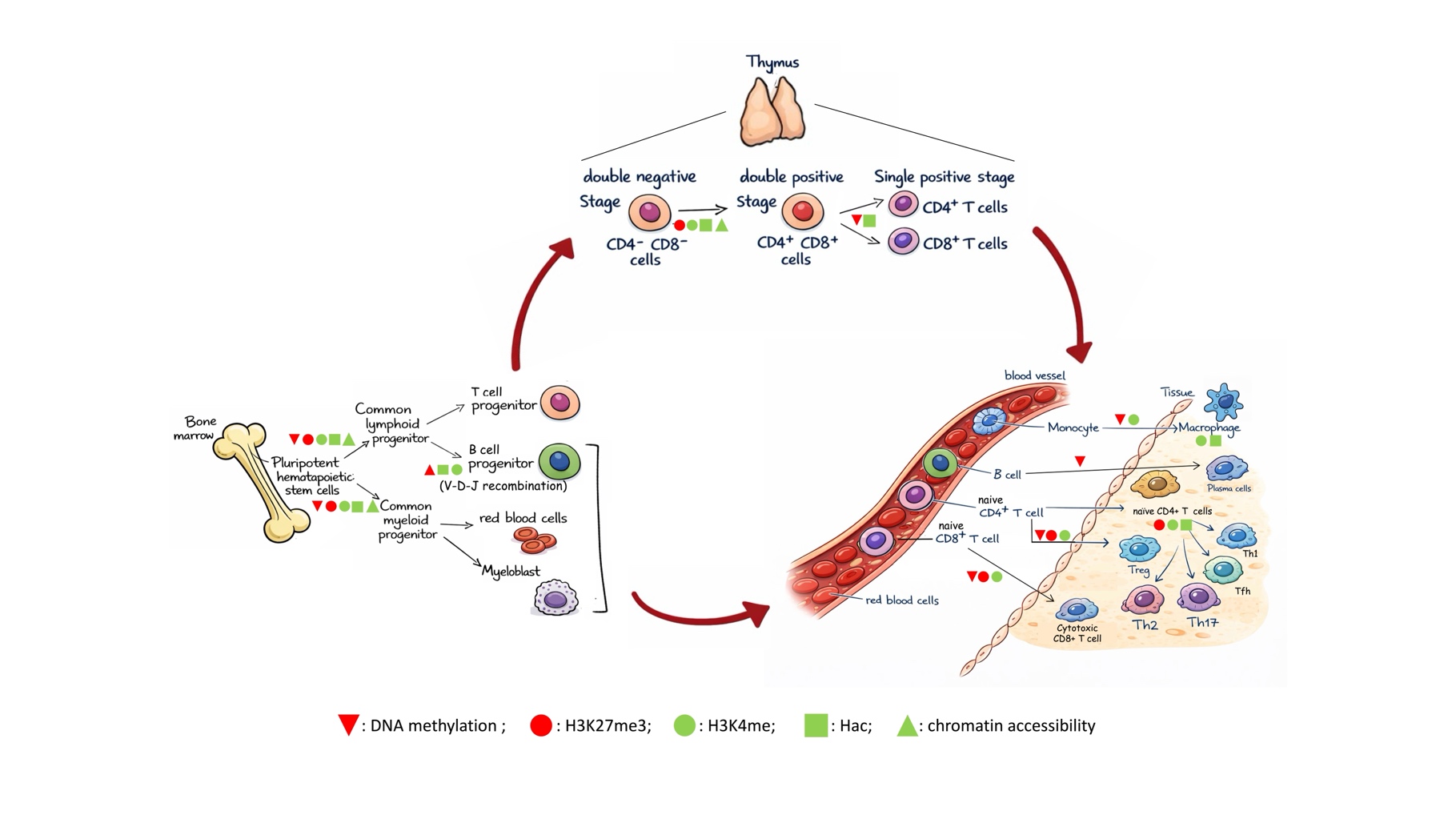
**

**Supplementary Figure 1: Epigenetic landscape changes along the development and differentiation of immune cells.** Hematopoiesis in the bone marrow, T cell maturation in the thymus, and differentiation of mature immune cells in the periphery together with changes in several epigenetic modifications are depicted. Red tringle: DNA methylation, red circle: H3K27 trimethylation, green circle: H3K4 methylation, green square: histone acetylation, green triangle: chromatic accessibility. The image was generated using OpenAI’s ChatGPT (https://chat.openai.com).


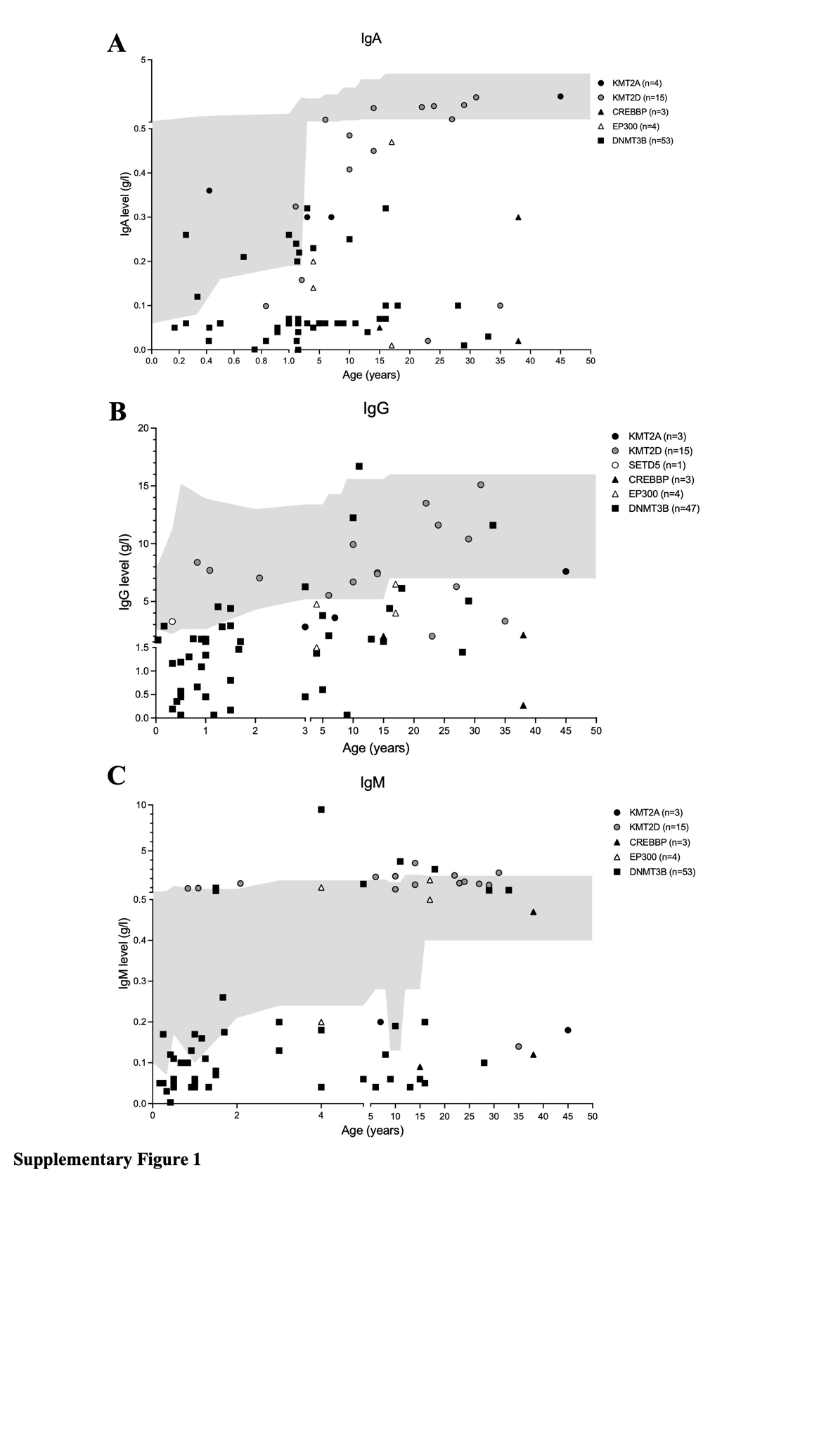


**Supplementary Figure 2:** Immunoglobulin levels in patients with different chromatinopathies and age-specific reference values. **(A)** IgA, **(B)** IgG, and **(C)** IgM levels are illustrated in individuals with various chromatinopathies together with normal range of immunoglobulin concentrations for each age group represented as grey areas. Each symbol shows an immunoglobulin level of an individual. Each gene is represented with a different shape and shade. Histone methyltransferases (KMT2A, KMT2D, SETD5) with circles, histone acetyltransferases (CREBBP, EP300) with triangles, and DNA methyltransferase (DNMT3B) with squares are displayed. CREBBP: CREB-binding protein; DNMT: DNA methyltransferase; EP300: E1A-associated protein p300; Ig: immunoglobulin; KMT: lysine methyltransferase; SET: Su(var), Enhancer of zeste, Trithorax; SETD: SET domain-containing protein

## Supplementary Tables

**Supplementary Table 1:** Detailed research strategy together with search terms and filter terms are listed.

| A Major immunoglobulins (g/l) | | | | | | | | | | | |
| --- | --- | --- | --- | --- | --- | --- | --- | --- | --- | --- | --- |
|  | 0-2 wks | 0.5-4 mo | 4-6 mo | 6-12 mo | 1-2 y | 2-3 y | 3-6 y | 6-9 y | 9-12 y | 12-16 y | Adult |
| IgA | <0.16 | 0.06  -  0.57 | 0.08  -  0.90 | 0.16  -  0.98 | 0.19  -  1.1 | 0.19  -  2.3 | 0.55  -  2.2 | 0.54  -  2.5 | 0.62  -  3.0 | 0.70  -  3.6 | 0.70  -  4.0 |
| IgG | 6.5  -  12.6 | 2.6  -  7.8 | 2.2  -  11.3 | 2.6  -  15.2 | 2.6  -  13.9 | 4.3  -  13.0 | 5.2  -  13.4 | 5.2  -  14.3 | 5.2  -  15.6 | 5.2  -  15.6 | 7.0  -  16.0 |
| IgM | 0.03  -  0.24 | 0.10  -  0.55 | 0.07  -  0.65 | 0.17  -  1.2 | 0.10  -  0.87 | 0.21  -  0.87 | 0.24  -  1.8 | 0.28  -  1.9 | 0.13  -  1.6 | 0.28  -  2.4 | 0.40  -  2.3 |

| B IgG subclasses (g/l) | | | | | | | | | | | | | |
| --- | --- | --- | --- | --- | --- | --- | --- | --- | --- | --- | --- | --- | --- |
|  | 0-1 mo | 1-4 mo | 4-6 mo | 6-12 mo | 1-1.5 y | 1.5-2 y | 2-3 y | 3-4 y | 4-6 y | 6-9 y | 9-12 y | 12-16 y | Adult |
| IgG1 | 2.4  -  10.6 | 1.8  -  6.7 | 1.8  -  7.0 | 2.0  -  7.7 | 2.5  -  8.2 | 2.9  -  8.5 | 3.2  -  9.0 | 3.5  -  9.4 | 3.7  -  10.0 | 4.0  -  10.8 | 4.0  -  11.5 | 3.7  -  12.8 | 4.9  -  11.4 |
| IgG2 | 0.87  -  4.1 | 0.38  -  2.1 | 0.34  -  2.1 | 0.34  -  2.3 | 0.38  -  2.4 | 0.45  -  2.6 | 0.52  -  2.8 | 0.63  -  3.0 | 0.72  -  3.4 | 0.85  -  4.1 | 0.98  -  4.8 | 1.06  -  6.1 | 1.50  -  6.4 |
| IgG3 | 0.14  -  0.55 | 0.14  -  0.70 | 0.15  -  0.80 | 0.15  -  0.97 | 0.15  -  1.07 | 0.15  -  1.13 | 0.14  -  1.20 | 0.13  -  1.26 | 0.13  -  1.33 | 0.13  -  1.42 | 0.15  -  1.49 | 0.18  -  1.63 | 0.20  -  1.10 |
| IgG4 | 0.039  -  0.56 | 0.022 -  0.36 | 0.017 -  0.23 | 0.012  -  0.43 | 0.011  -  0.62 | 0.011  -  0.79 | 0.012  -  1.06 | 0.015 -  1.27 | 0.017 -  1.58 | 0.023 -  1.89 | 0.030 -  2.1 | 0.035  -  2.3 | 0.080  -  1.40 |

| C Lymphocyte subpopulations (x10^9^/l) | | | | | | | | | | |
| --- | --- | --- | --- | --- | --- | --- | --- | --- | --- | --- |
|  | Neo-natal | 1 wk-  2 mo | 2-5 mo | 5-9 mo | 9-15 mo | 15-24 mo | 2-5 y | 5-10 y | 10-16 y | Adult |
| Lymphocytes | 0.7  -  7.3 | 3.5  -  13.1 | 3.7  -  9.6 | 3.8  -  9.9 | 2.6  -  10.4 | 2.7  -  11.9 | 1.7  -  6.9 | 1.1  -  5.9 | 1.0  -  5.3 | 1.0  -  2.8 |
| CD19+  B lymphocytes | 0.04  -  1.1 | 0.6  -  1.9 | 0.6  -  3.0 | 0.7  -  2.5 | 0.6  -  2.7 | 0.6  -  3.1 | 0.2  -  2.1 | 0.2  -  1.6 | 0.2  -  0.6 | 0.1  -  0.5 |
| CD3+  T lymphocytes | 0.6  -  5.0 | 2.3  -  7.0 | 2.3  -  6.5 | 2.4  -  6.9 | 1.6  -  6.7 | 1.4  -  8.0 | 0.9  -  4.5 | 0.7  -  4.2 | 0.8  -  3.5 | 0.7  -  2.1 |
| CD3+/CD4+  T lymphocytes | 0.4  -  3.5 | 1.7  -  5.3 | 1.5  -  5.0 | 1.4  -  5.1 | 1.0  -  4.6 | 0.9  -  5.5 | 0.5  -  2.4 | 0.3  -  2.0 | 0.4  -  2.1 | 0.3  -  1.4 |
| CD3+/CD8+  T lymphocytes | 0.2  -  1.9 | 0.4  -  1.7 | 0.5  -  1.6 | 0.6  -  2.2 | 0.4  -  2.1 | 0.4  -  2.3 | 0.3  -  1.6 | 0.3  -  1.8 | 0.2  -  1.2 | 0.2  -  0.9 |
| CD3−/CD16+/CD56+  NK cells | 0.1  -  1.9 | 0.2  -  1.4 | 0.1  -  1.3 | 0.1  -  1.0 | 0.2  -  1.2 | 0.1  -  1.4 | 0.1  -  1.0 | 0.09  -  0.9 | 0.07  -  1.2 | 0.09  -  0.6 |

**Supplementary Table 2:** Age-matched references for immunological laboratory data are listed. **(A)** Major immunoglobulins and **(B)** IgG subclasses calculated as g/l are derived from Sanquin, 2014 (www.sanquin.org) ^49^. **(C)** Lymphocyte subpopulation numbers shown as x10^9^/l are derived from Comans-Bitter et al, 1997 ^48^.

**Supplementary Table 3:** The immunological and genetic findings in patients with chromatinopathies included in the scoping review are summarized in this table. Each patient is presented with the affected gene, age at evaluation, gammaglobulin levels (IgG, IgG subclasses, IgA, IgM), lymphocyte subsets (T, B, and NK cells), nucleotide and protein variants, mutation classification, predicted or reported functional protein consequences, and associated infectious and immunopathological manifestations. Patients with cross-referenced are shown in bold. AIHA: autoimmune hemolytic anemia; CID: combined immune deficiency; CVID: common variable immune deficiency; d/o: days old; GLILD: granulomatous–lymphocytic interstitial lung disease; IVIG: intravenous immunoglobulin; ITP: immune thrombocytopenia; m/o: months old; NR: not reported; PID: primary immunodeficiency; SCID: severe combined immunodeficiency; y/o: years old.
